# Rheological Insights: The Role of Divalent Ions on the Stability and Phase Behavior of Oil–Water Emulsions

**DOI:** 10.1021/acsomega.6c01366

**Published:** 2026-09-29

**Authors:** Janett Montaño-Salazar, A. Paulina Gómora-Figueroa, Roberto Zenit, Rosa María Mariscal-Romero

**Affiliations:** † División de Ingeniería en Ciencias de la Tierra, Facultad de Ingeniería, 7180 Universidad Nacional Autónoma de México, Circuito Exterior, Ciudad Universitaria, 04510 Ciudad de México, Mexico; ‡ 6752 School of Engineering, Brown University, 184 Hope St, Providence, Rhode Island 02912, United States

## Abstract

This study explores the rheological insights into how divalent ions, specifically calcium (Ca^2+^) and magnesium (Mg^2+^), influence the stability and phase behavior of oil-in-water (O/W) emulsions. O/W emulsions are employed for different purposes in the petroleum industry, including drilling muds, viscosity-reducing agents, enhanced oil recovery (EOR), and transportation of crude oil. We investigated the effects of salinity concentration, time, and surfactant dosage on emulsion properties. Eighteen stable emulsions were prepared using CaCl_2_ and MgCl_2_ brines at 0.5% (group **A**) and 5.0% v/v (group **B**) concentrations of a commercial anionic surfactant, Hercoten, (from a carboxylate family). Our findings demonstrate that brine composition significantly affects emulsion phase inversion, rheological behavior, and viscosity; CaCl_2_ emulsions reached viscosities of up to 240 cP at 25,000 ppm (day 7), up to one order of magnitude higher than MgCl_2_ systems. Furthermore, we identified phase inversion in CaCl_2_ systems and evidence of multiple emulsions (O/W/O) in MgCl_2_ systems, both at 8000 ppm and 5.0% v/v surfactant. To complement the experimental analysis, we developed a custom Python-based tool, ROJA, for automated image analysis and statistical characterization of emulsion droplets. We suggest that intermolecular forces and increased friction due to electrostatic interactions between Ca^2+^ and the anionic surfactant led to higher viscosities and smaller, more concentrated droplets (as low as 54.27 μm at 25,000 ppm) at higher salinities. In contrast, Mg^2+^ emulsions showed lower viscosities due to larger droplets (up to 127.87 μm at 25,000 ppm), resulting in fewer droplet–droplet interactions. The presence and concentration of divalent ions are therefore critical for the performance of O/W emulsions in petroleum industry applications.

## Graphical abstract

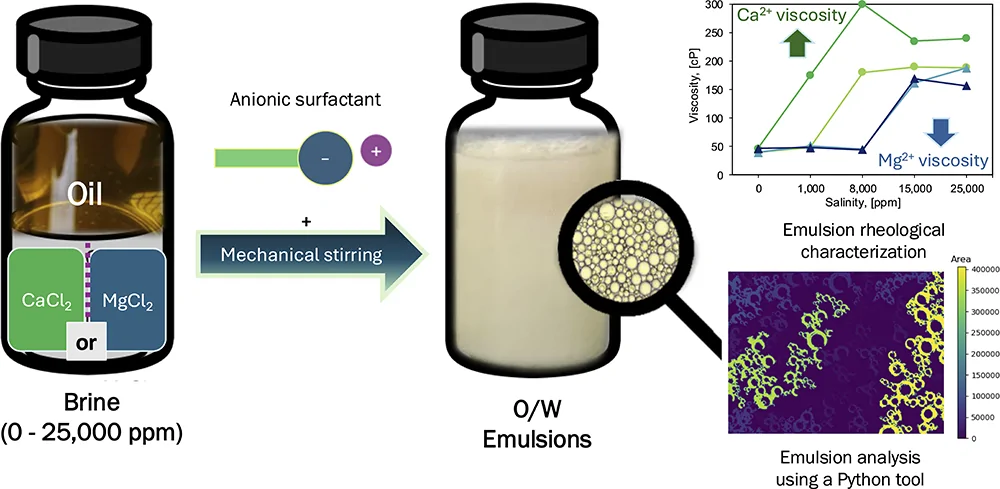

## 1 Introduction

Numerous mature oil fields encounter flow assurance challenges due to water production, often resulting in the formation of water-in-oil (W/O) emulsions that significantly impact the handling, conditioning, and processing of hydrocarbons.^1–3^ Oil’s viscosity is pivotal in mature oil fields as it directly influences reservoir recovery and productivity. Although density and viscosity are not directly correlated, a decrease in °API usually corresponds to an increase in viscosity. Consequently, transporting heavy oils through porous media and pipelines demands additional energy, such as heat or diluents, to ensure acceptable flow rates.^4^

The formation of oil-in-water (O/W) emulsions is one of the few viable methods for transporting heavy oils, with the potential to reduce viscosity to 50-200 cP. Additionally, emulsion technology offers an avenue to enhance residual oil recovery from mature fields that may not be efficiently recovered through traditional methods involving heat or diluents.^5^ Emulsions suitable for pipeline transport, containing up to 75% crude, have demonstrated a viscosity reduction of near to two orders of magnitude. This economic and operational preference aims to maximize oil transportation while minimizing the water content.^4,6–10^

The O/W emulsions find diverse applications in the petroleum industry, including use in drilling muds, viscosity-reducing agents, and enhanced oil recovery (EOR) additives; for example, their injection into reservoirs to enhance mobility control of heavy oils, improve conformance, and increase the recovery factor of mature fields. Blocking more permeable zones with emulsion drops can improve reservoir sweep, resulting in substantial additional recoveries compared to conventional water flooding.^11,12^ O/W emulsion flooding may be more effective than some chemical EOR techniques, such as polymer flooding.^13^

The stability of emulsions hinges on various factors, including the viscosity of the continuous phase, the volume fraction of components, droplet size, interfacial tension, and the density of the different components.^14^ External factors such as inorganic species, temperature, droplet-size distribution, brine pH, and composition also influence emulsion stability.^15^ For instance, many studies have reported that salinity strongly influences emulsions’ stability.^2,16–21^ Moradi et al. 2011 ^22^ concluded that low ionic strength favors the stability of water/oil emulsions, and Wang et al. 2012,^23^ presented results consistent with these findings. In contrast, Perles et al. 2012 ^24^ observed that the presence of salts increases the stability of water/oil emulsions up to a critical value. Castro Nóbrega et al. 2022,^25^ reported that higher salt concentrations result in larger droplets for O/W emulsions, whereas for W/O emulsions, smaller droplets are formed, leading to more stable emulsions, consistent with the findings of Mohamed Reyani et al. 2023.^19^ Additionally, higher salt concentrations in the water phase can enhance the stability of O/W emulsions, potentially due to ion barriers between oil droplets and the continuous water phase.^26,27^

The effect of divalent ions has been studied for more complex systems, such as that reported by Mansouri et al. 2025,^20^ whose study introduces a targeted chemical method that leverages the synergistic interplay between specific brine ions, calcite particles, asphaltene molecules, and cationic surfactants. High concentrations of magnesium or calcium ions promote the formation of rigid asphaltene complexes, which stabilize emulsions and hinder oil mobilization, while divalent ions also enhance the accumulation of the cationic surfactant at the oil-water interface, increasing the electrostatic repulsion between droplets. This complex interplay underscores the critical role of the ionic composition in determining emulsion stability and effective oil recovery strategies.

Related to this, it has been reported that increasing the salinity up to 8,000 ppm reduces the average droplet size, promoting better dispersion of the aqueous phase and enhancing emulsion stability; however, beyond this threshold, the droplet size increases and the emulsion become less stable. This trend can be explained by the Gibbs adsorption isotherm: as salt concentration increases, the oil’s capacity to adsorb salt at the oil–water interface is eventually exceeded, causing salt to accumulate preferentially in the bulk aqueous phase rather than at the interface. As a result, the excess interfacial area becomes negative, leading to decreased emulsion stability.^28^ Nonetheless, chemical reactions between salt ions and surfactants can affect emulsion stability, as excess ions may lead to surfactant precipitation and hinder performance.^26,29^

Recent reports demonstrate that divalent ions, particularly calcium (Ca^2+^) and magnesium (Mg^2+^), play a crucial role in surfactant performance, emulsion formation, and stability.^19,20,25,29–32^ For instance, the phase behavior of anionic surfactant systems is more sensitive to changes in Ca^2+^ and Mg^2+^ concentrations than in Na^+^ (monovalent cations), a phenomenon that becomes more pronounced at lower surfactant/TDS (total dissolved solids) concentrations.^33^ Anionic surfactants with modified structures, designed to withstand high temperatures and salinity, have demonstrated greater resistance to these ions.^34^

Although many laboratory studies have examined the conditions and components described above to understand the emulsion formation and stability, most of this work has focus on W/O emulsions, which represent one of the significant challenges in the petroleum industry during recovery, treatment, and transportation of crude oil, reducing oil quality, affecting flow behavior, and ultimately leading to flow assurance problems.^35^ Some studies suggest that Ca^2+^ and Mg^2+^ ions interact significantly with the emulsifiers used in drilling fluids, altering their functional properties and stabilizing W/O emulsions.^21,25,36^ Castro Nóbrega et al. 2022,^25^ suggested that divalent ions can interact at the droplet interface as counterions, due to the droplets’ positive electrical charge, altering the physical properties of the interfacial film and thereby increasing the solubility of the oil phase. Similarly, Hao et al. 2023,^30^ evaluated the effect of pH and cations on the formation and stability of crude oil emulsions, focusing on the formation process, droplet size, and the stability mechanisms under different conditions; their results showed that the emulsification potential of cations followed the order: CaCl_2_ > MgCl_2_ > NaCl, and the authors suggested that divalent ions form chemical bonds between emulsion droplets. Kazemzadeh et al. 2019 ^31^ studied W/O emulsification of crude oil at various pressures and salinities, showing that the type and concentration of cations present in the aqueous phase govern the emulsion stability: at lower salinities, W/O emulsion stability increases, decreasing as salt concentration rises, and divalent cations form more stable emulsions than monovalent ions at high pressures.

Despite this growing body of work on W/O systems, there is still a lack of understanding of the stability mechanisms governing O/W emulsions under different conditions. The present work aims to evaluate the effects of Ca^2+^ and Mg^2+^ ions on the formation and stability of the O/W emulsions. Other parameters considered in this work include salinity, time, and surfactant concentration, since emulsification involves several concurrent processes (droplet breakup, surfactant adsorption, and droplet collisions) that occur repeatedly, over very short time scales.^37^ Understanding the complex interplay of factors that influence emulsion stability is critical for the successful application of emulsions across various petroleum industry processes.

It is important to note that, given the complexity of working with live crude oil and reservoir fluids under controlled laboratory conditions, this study relies on a model oil system rather than real crude oil, which constitutes a limitation when extrapolating these findings directly to field-scale operations. Nevertheless, the relevance of the model system employed here to actual field conditions is supported by several considerations. First, synthetic, mineral, and vegetable oils have been widely used as proxies for crude oil in fundamental emulsion studies, allowing controlled and reproducible evaluation of interfacial phenomena without the compositional variability inherent to crude oil.^38–44^ Second, the surfactant concentrations and brine compositions tested in this work (0–25,000 ppm) fall within the range of conditions encountered in EOR operations and produced water handling. Third, the ionic interactions between anionic surfactants and divalent ions (Ca^2+^, Mg^2+^) identified in this study are consistent with those reported in more complex, crude-oil-based systems.^19,20,25,31,40,45,46^ Taken together, these considerations indicate that, while direct validation with reservoir fluids remains necessary, the model system used here provides mechanistic insights into the role of divalent ions in O/W emulsion stability that are expected to be transferable to real crude oil systems.

## 2 Materials and Methods

### 2.1 Materials

All reagents were obtained from Sigma-Aldrich and used without further purification. The NaCl, CaCl_2_ and MgCl_2_ brines (0, 1000, 8000, 15000, and 25000 ppm) were prepared using distilled water and the corresponding salt; NaCl (0.000, 0.017, 0.137, 0.257, and 0.428 M), MgCl_2_·6H_2_O (0.000, 0.005, 0.039, 0.074, and 0.123 M), or CaCl_2_·2H_2_O (0.000, 0.007, 0.054, 0.102, and 0.170 M), all salts were reagent grade (purity ≥ 99%). Also, we used distilled water (0 ppm salinity) to prepare emulsions as a control experiment. Brines were employed to prepare three groups (10 each) of 70/30 ratio emulsions, by mixing 70 mL of oil (ρ = 0.918 g/mL, μ = 72.7 cP; composed mainly of triglycerides rich in oleic acid, (C_57_H_104_O_6_)) and 30 mL of brine.

The commercial anionic surfactant used in this study, referred to as **H**, is Hercoten (ρ = 0.923 g/mL, pH = 13.5, CMC = 0.43%), supplied by Cosmopolita. The surfactant belongs to the carboxylate family of anionic surfactants. The infrared and ^1^H/^13^C NMR spectra are shown in Figures S11–S13. 0.5% v/v of H was added to the first group (**A**) of emulsions and 5.0% v/v to the second group (**B**).

### 2.2 Preparation of Emulsions

This section details the procedure for 70/30 ratio systems with Na^+^, Ca^2+^, and Mg^2+^ brines. It is worth noting that we studied 50/50 and 30/70 oil-brine ratios; however, the 70/30 ratio led to the most stable emulsions overall. Emulsions were prepared by vigorously mixing 30 mL of brine plus surfactant **H** (0.5% −**A**– or 5.0% v/v −**B**−) and 70 mL of oil using the IKA RW20 Digital homogenizer at a rate of 700 r/min for 15 min at room temperature (298 K). The emulsions were allowed to equilibrate on the bench and kept under their initial preparation conditions at ambient temperature (298 K), and the glass bottle was sealed. We studied the properties of the resulting systems from day 0 to day 7. [Day 0 corresponds to the day the emulsion was prepared.]

### 2.3 Stability of Emulsions

The stability of the emulsions was measured qualitatively by observing phase separation over 20 days. All Mg^2+^ emulsions from group **A** were stable, but phase separation started 200 min after preparation (day 0), therefore, we characterized these emulsions immediately after formulation. The Ca^2+^ emulsions from the same group (**A**) showed more stability, since the homogeneous liquid phase remains up to day 9 (see Figures 1 and S3). Since NaCl systems (group **A**) were unstable, we only characterized the 1,000 ppm emulsion (5.0% v/v at **H** dosage), which resulted in a shear-thinning O/W system at day 0. No further characterization was possible; thus, we did not discuss the data using this ion.

**Figure 1:**
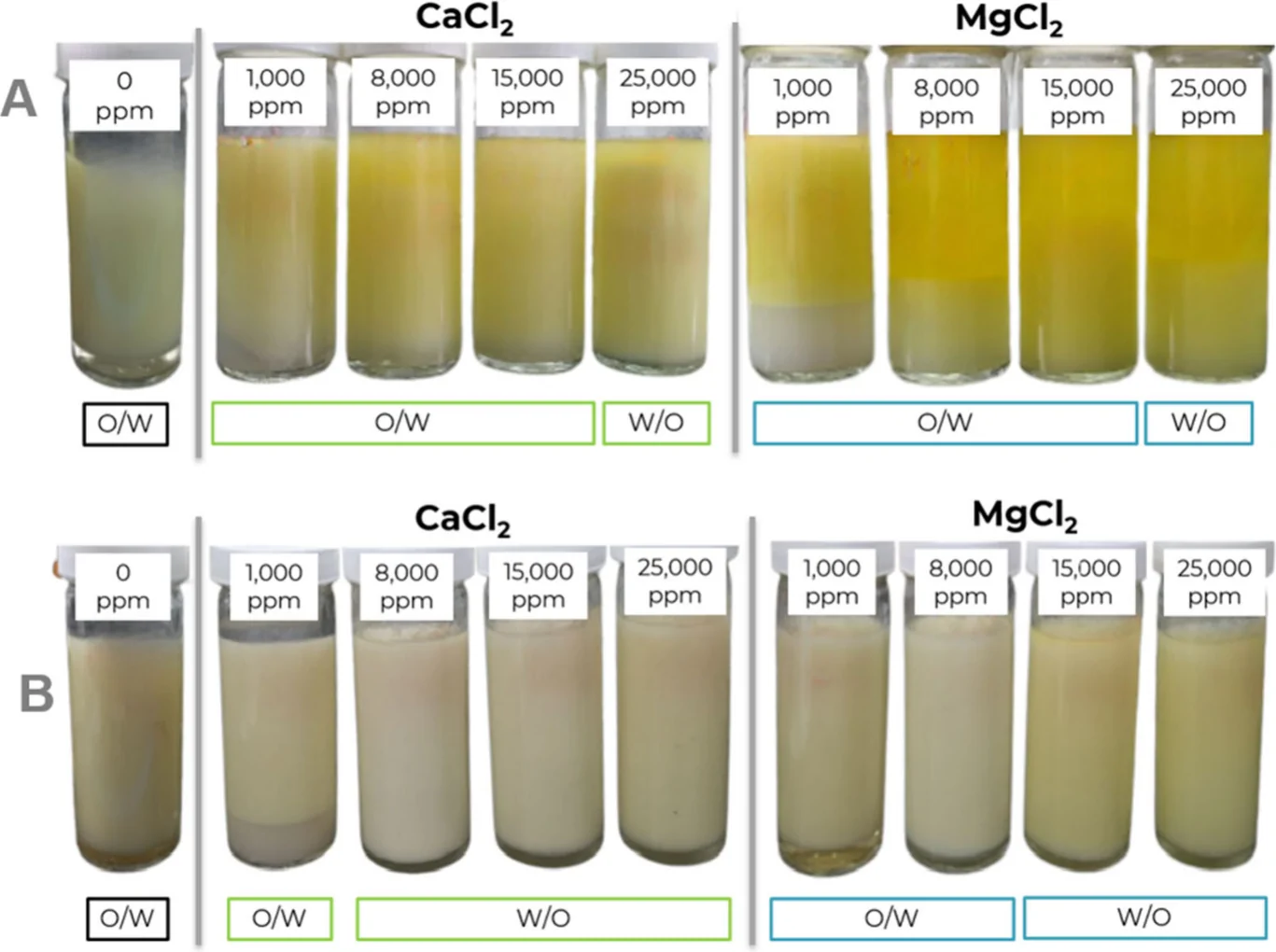
Emulsions formed with 0.5% v/v of **H** (**A**) and 5.0% v/v (**B**) on day 0, after characterization (∼250 min).

Group **B** emulsions were more stable than group **A**. For instance, the Mg^2+^ systems remain steady over time (day 20), especially at lower salinities (0, 1,000 and 8,000 ppm). The Ca^2+^ emulsions from this group (**B**) were the most stable systems, overall. For consistency, we characterized the emulsions up to day 7, although some of the low salinity systems remained unchanged for more than 20 days (see Figures S3 and S4).

### 2.4 Surface Tension

The surface tension was measured using a SITA tensiometer DynoTester+, based on the bubble pressure method for measuring the dynamic surface tension. The bubble pressure method is based on passing an air stream is led through a capillary into the process liquid. A sensor measures the pressure needed to generate a bubble. According to the Young–Laplace equation, the pressure difference, ΔP = ΔP_max_ – ΔP_min_, observed during the bubble’s lifetime, is proportional to the surface tension (mN/m), σ. We used a beaker to measure the surface tension of the brine solutions containing surfactant **H** (0.5% and 5.0%) and set the lifetime at 50 ms.

### 2.5 Characterization of Emulsions

#### 2.5.1 Emulsion Type

Immediately after preparing the emulsion, we used the drop test, a qualitative method to infer the type of system formed. Thus, in a 5 mL vial with distilled water, one drop of emulsion was added; if the drop dispersed into the water, then the system was inferred to be an oil-in-water (O/W) emulsion. In the opposite case, if the drop remained suspended in water, the resulting system was inferred to be a water-in-oil (W/O) emulsion. Emulsion stability against creaming and coalescence was determined by monitoring the position of the oil, water, and emulsion interfaces over time (bottle test).

#### 2.5.2 Optical Microscopy and Image Analysis

An OMAX A3550U microscope was used to capture images of the emulsion drops, using a syringe to place a drop on a slide covered with a coverslip. Micrographs were taken at magnifications of 10x and with the Toup View microscope software. Samples for microscopic analysis systematically were collected from near the top of the glass bottle with a pipet and placed on a glass slide. The emulsion droplet size distribution was analyzed over time using micrographs and an image processing program, that we developed (ROJA). Although open-source software such as ImageJ is widely used for droplet analysis, it presents limitations under noisy imaging conditions. It frequently misidentifies gaps or suspended particles as droplets, requiring manual correction and pre-processing steps. To address these issues, ROJA integrates specialized libraries and filters for the accurate segmentation and characterization of droplets in complex emulsions. This tool enables automated extraction of key metrics, including droplet area and radius, along with descriptive statistics (mean, median, mode, and standard deviation), outputted in Excel-compatible format (see Table S7). Compared with ImageJ, ROJA generates cleaner and more accurate binary masks, effectively discriminating droplets from background noise and particulate impurities. Additionally, the software produces spatial droplet distribution heatmaps (Figure S7), facilitating visual interpretation of the size heterogeneity across the emulsion.

### 2.6 Viscosity and Rheological Behavior

A Brookfield DvNext viscometer was used to measure the viscosity (μ) [cP] and the shear rate (*γ̇*) [1/s] from 30 to 250 revolutions per minute (rpm) for 20 seconds at each shear rate. The following equation was used to determine the shear stress (τ) [mPa].

τ=γ̇·μ

The rheological behavior was obtained by applying Oswald’s law (power-law) τ = *K*(*γ̇*)^*n*^, where τ is in Pa, *K* consistency index (apparent viscosity) is in [Pa·s], (*γ̇*) is in [1/s], and *n* is the fluency index; if *n* = 1 the fluid is *Newtonian*; if *n* < 1, the fluid is *shear-thinning*, and if *n* > 1, the fluid is *shear-thickening*.

## 3 Results and Discussion

As expected, surface tension decreased with increasing the surfactant concentration, favoring emulsion formation and stability (Figures S1 and S2). Surface tension for brines using 0.5% v/v concentration of **H** increased with salinity, ranging from 52.2 mN/m (0 ppm) to 71.9 mN/m (25 000 ppm). When the surfactant concentration was increased to 5.0% v/v, the surface tension ranged from 41 mN/m (1000 ppm) to 63 mN/m (25,000 ppm). We used distilled water as the control brine, with a surface tension of 72.1 mN/m.

Figure 1 shows the Ca^2+^ and Mg^2+^ emulsions prepared with 0.5% v/v (**A**) and 5.0% v/v (**B**) of **H** on day 0, using a 70/30 oil–brine ratio. See Figure S3 for day 20. Figures 2 and 3 show the effect of salinity at a constant shear rate of 4.17 s^–1^. Tables 1 and 2 exhibit the type of emulsion, fluency index “n”, and the rheological behavior for groups **A** and **B**, on day 0, respectively. Shear stress (Pa) was determined using the power law and the shear rate (s^–1^) for MgCl_2_ and CaCl_2_ emulsions over time, within a range from 0.5 to 4.17 s^–1^.

**Figure 2:**
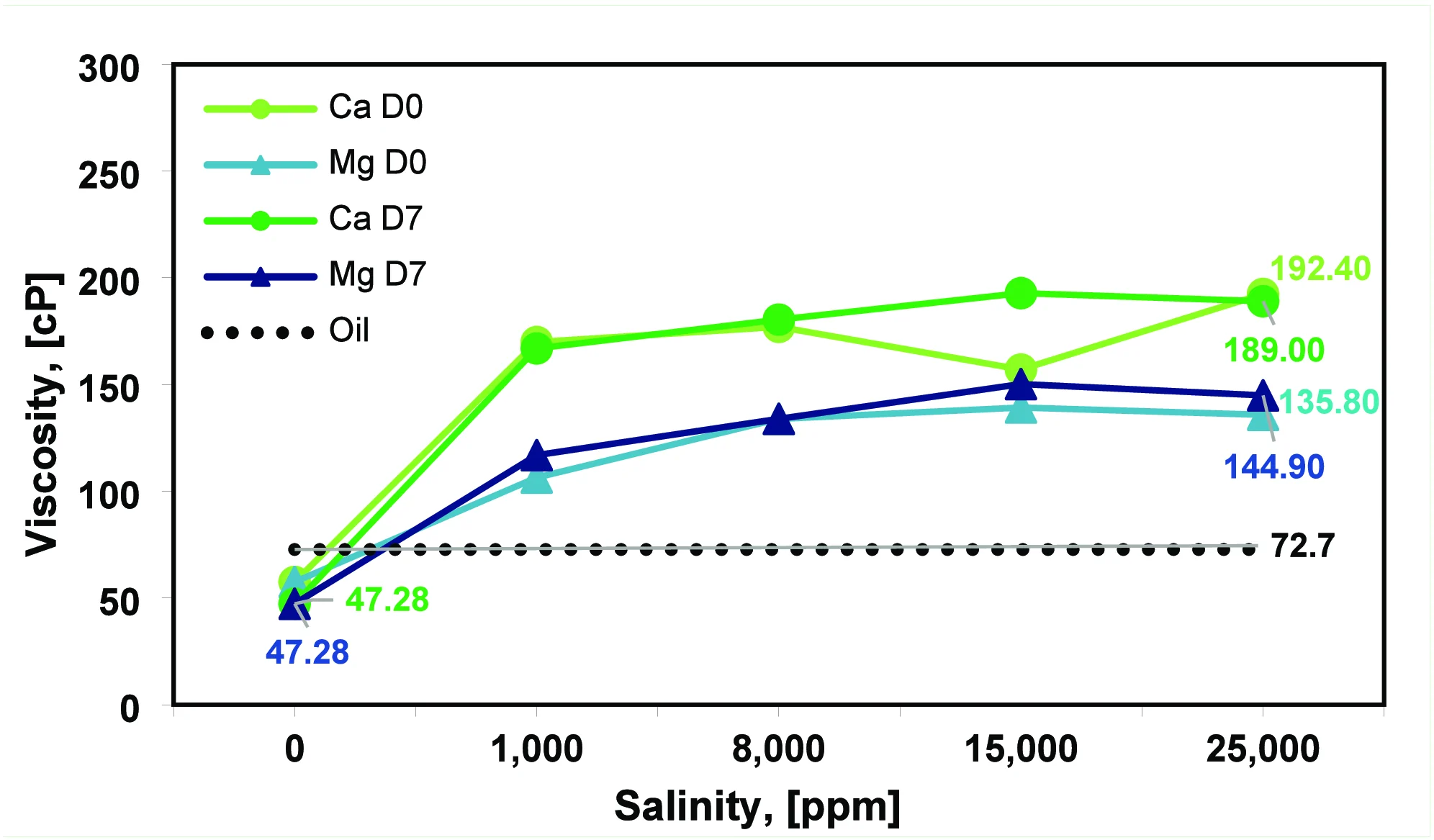
Viscosity vs salinity for emulsions prepared with CaCl_2_ (green) and MgCl_2_ (blue) for **A**. The dotted line represents the viscosity of the oil employed to prepare the emulsions. Lighter colors correspond to day 0 results. Darker colors correspond to day 7 results.

**Figure 3:**
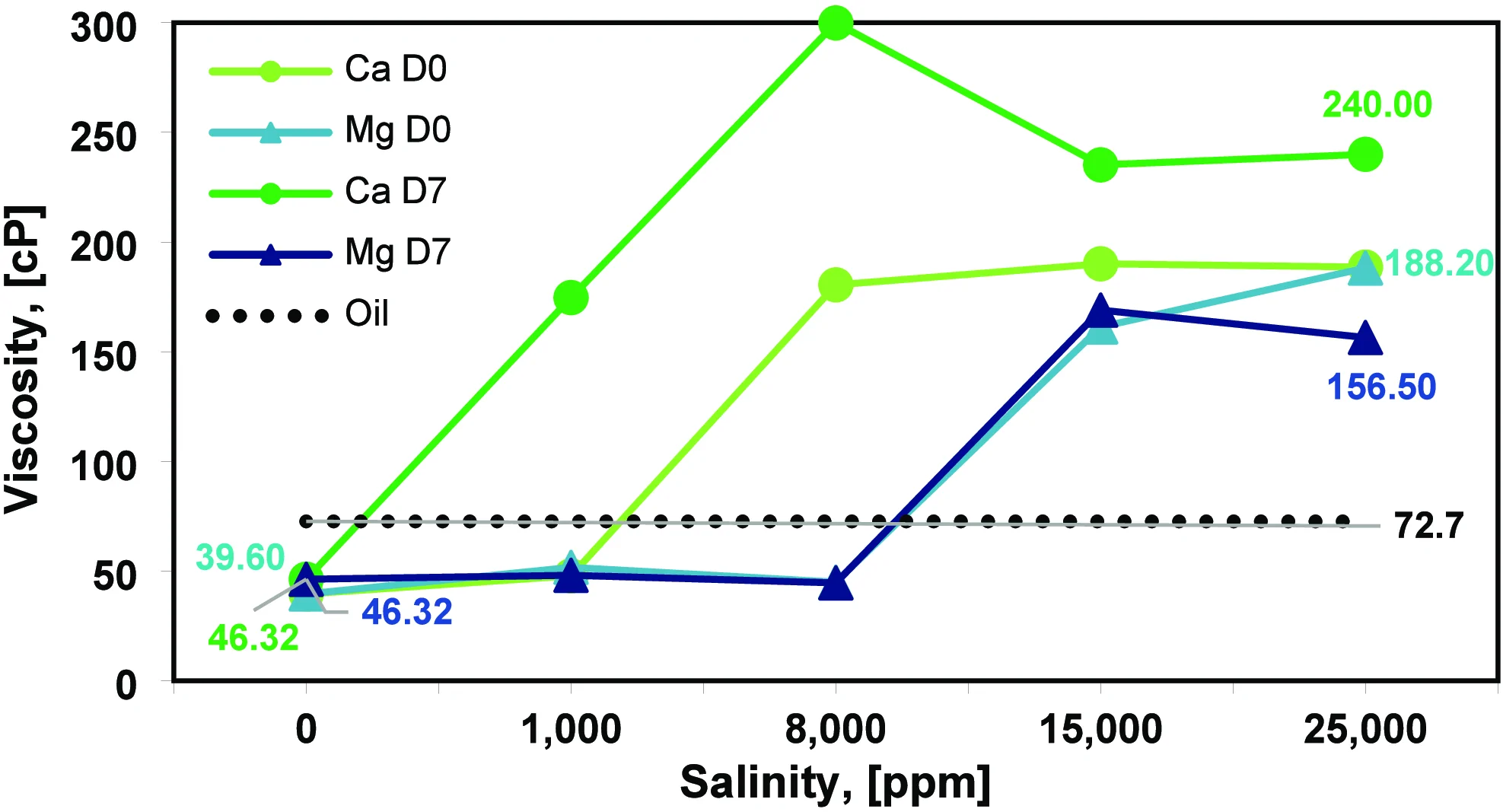
Viscosity vs salinity for emulsions prepared with CaCl_2_ (green) and MgCl_2_ (blue) for **B**. The dotted line represents the viscosity of the oil employed to prepare the emulsions. Lighter colors correspond to day 0 results. Darker colors correspond day 7 results.

**Table 1:** Type of Emulsion, Fluency Index “n”, and Rheological Behavior with Salinity for Group **A** (CaCl_2_ and MgCl_2_ at 0.5% v/v of Surfactant) on Day 0^a^

	CaCl_2_ 0.5% D0			MgCl_2_ 0.5% D0		
salinity ppm	emulsion type	rheological behavior	*n*	emulsion type	rheological behavior	*n*
0	O/W	shear-thinning	0.566	O/W	shear-thinning	0.566
1,000	O/W	shear-thickening	1.057	O/W	shear-thickening	1.043
8,000	O/W	shear-thickening	1.035	O/W	shear-thinning	0.918
15,000	O/W	shear-thickening	1.035	O/W	shear-thinning	0.896
25,000	W/O	shear-thinning	0.982	W/O	shear-thinning	0.942

a See Table S1 in the supporting information for day 7 results.

**Table 2:** Type of Emulsion, Fluency Index “*n*”, and Rheological Behavior with Salinity for Group **B** (CaCl_2_ and MgCl_2_ at 5.0% v/v of Surfactant) on Day 0^a^

	CaCl_2_ 5.0% D0			MgCl_2_ 5.0% D0		
salinity ppm	emulsion type	rheological behavior	*n*	emulsion type	rheological behavior	*n*
0	O/W	shear-thinning	0.738	O/W	shear-thinning	0.738
1,000	O/W	shear-thinning	0.653	O/W	shear-thinning	0.652
8,000	W/O	shear-thinning	0.854	O/W	shear-thinning	0.667
15,000	W/O	shear-thinning	0.797	W/O	shear-thinning	0.747
25,000	W/O	shear-thinning	0.819	W/O	shear-thinning	0.756

a See Table S2 in the supporting information for day 7 results.

For CaCl_**2**_ systems in group **A**, most of the emulsions were O/W and exhibited shear-thickening behavior. On day 7, we observed that the rheological behavior of 1,000 ppm emulsion changed from shear-thickening (day 0) to shear-thinning (day 7), see Tables 1 and S1, respectively. Increasing surfactant concentration to 5.0% v/v (**B**) resulted mainly in W/O emulsions for 8,000 to 25,000 ppm. The rheological behavior of all of the emulsions was shear thinning. (see Tables 2 and S2).

Most of the **MgCl_2_** systems in group **A** resulted in an O/W and exhibited a shear-thinning behavior at day 0 (Table 1). Nevertheless, we identified (in the micrographs) that on day 0, the 8,000 ppm emulsion was a multiple system (O/W/O) at high surfactant concentrations (**B**), suggesting that a phase inversion occurs at this salinity, explaining the change in emulsion type at higher ionic strength. As mentioned before, Mg^2+^ systems were less stable than the Ca^2+^ systems (section 2.3). For group **A**, the emulsions were regenerated on day 7 by mixing the phases at 700 r/min for 15 min at room temperature. Although emulsions were less stable, and phase separation was observed 15 min after re-emulsification, the emulsion type and rheological behavior remained similar to day 0 for all salinities; however, at 1,000 ppm, the rheological behavior changed from shear-thickening to shear-thinning, see Table S1.

In general, for both groups (**A** and **B**), the higher the salinity, the greater the viscosity. At 0.5% v/v (**A**) surfactant concentration viscosity was lower than 200 cP for all the emulsions. Only at 0 ppm the viscosity was lower (47.28 cP) than the oil phase (72.7 cP), which suggests that the ionic strength reduce the distance among droplets, resulting in greater interactions between the anionic surfactant and the divalent ions (Ca^2+^ and Mg^2+^). For group **B** (5.0% v/v), the CaCl_2_ emulsions were more viscous than MgCl_2_ by up to one order of magnitude (8,000 ppm). The viscosity trend endures over time; however, the viscosity increased for CaCl_2_, from day 0 to day 7 (188.2 and 240.0 cP at 25,000 ppm, respectively). MgCl_2_ systems did not show an increase in viscosity over time. See Figures 2 and 3. Nevertheless, the surfactant’s interaction with Ca^2+^ and Mg^2+^ differs in each case. For example, higher viscosity for the systems with CaCl_2_, in group **B**, could be attributed by significant interactions between Ca^2+^ ions and the emulsion droplets, resulting in an increase in friction, which is consistent with the findings reported previously,^19^ see Figure 3. A possible explanation is that Ca^2+^ ions interact simultaneously with two negatively charged droplet surfaces stabilized by the surfactant, specifically, with the carboxylate headgroups of the anionic surfactant at the oil-water interface, altering interfacial film rigidity. This electrostatic bridging promotes droplet aggregation (flocculation) without immediate coalescence, leading to the formation of a network-like structure that increases the apparent viscosity of the emulsion. Also, a more rigid interfacial film resists droplet deformation under shear, contributing to higher viscosity. Over time, these ionic interactions lead to progressive microstructural reorganization, such as compaction or partial coalescence, which is consistent with the observed viscosity increase.^47,48^

Yuan et al. 2023 ^49^ investigated the effects of calcium and magnesium ions on the stability of an anionic surfactant-emulsified asphalt. Their results showed that the addition of Ca^2+^ or Mg^2+^ to a salt-containing system induces aggregation between the head groups of the surfactant molecules, reducing the mobility of the water molecules surrounding the headgroup, which diminishes the thickness of the surfactant headgroup distribution along the *Z*-axis, weakening the stability of the anionic surfactant-emulsified asphalt. The authors concluded that the addition of Mg^2+^ reduces the stability of the emulsion, since Mg^2+^-emulsifier headgroup molecules bind to water molecules, making dissociation more difficult.^49^ Another example of this effect was reported by Mansouri et al. 2025,^20^ they evaluated a cationic surfactant, and compared emulsions prepared with magnesium and calcium brines. They found that calcium-enriched seawater stabilizes the emulsion film, whereas magnesium-enriched seawater reduces stability.

In the case of magnesium systems (see Figure 3), the viscosity of emulsions did not change significantly for **B** either, which suggests that this divalent ion interact less with charged droplet surfaces (carboxylate headgroups). The ionic radius of Mg^2+^ (72 pm) is smaller than that of Ca^2+^ (100 pm), resulting in a higher surface charge density;^50^ therefore, it is a better charge acceptor so it would be expected to bind more tightly to carboxylate groups; however, this causes it to hold onto its hydration shell of water molecules with great force. Ca^2+^ requires much less energy to shed its hydration layer and interact directly with the carboxylate group,^51^ resulting in low droplet concentration or droplet–droplet interactions, and a minimal change in viscosity.

The droplet radii for groups **A** and **B** ranged from 0.1 to 100 μm.^52^ CaCl_2_ and MgCl_2_ emulsions in group **A**, exhibited smaller droplet radii at lower salinities (0 to 8,000 ppm), and slightly increased with salinity (Figure 4); the trend remains over time. Previous reports^22,23^ showed that lower ionic strength conditions lead to more stable emulsions, due to smaller droplets, while high salinity reveals large droplets, resulting in less stability for emulsions, which is consistent with our findings.

**Figure 4:**
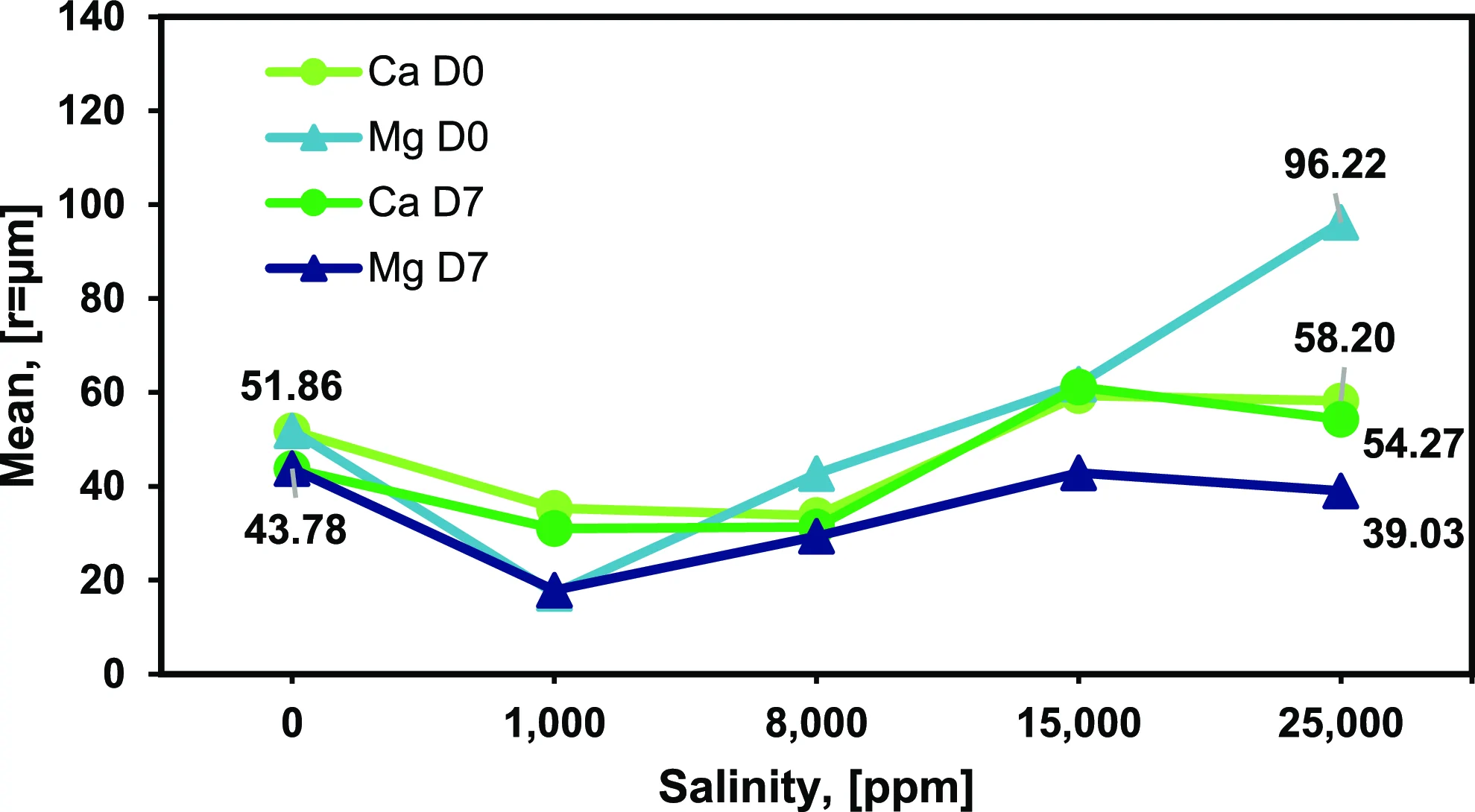
Mean radius vs salinity for 0.5% v/v surfactant concentration (**A**) over time. The droplets’ size of MgCl_2_ emulsions increased, on day 0, at higher salinity (25,000 ppm) compared to CaCl_2_ emulsions.

The MgCl_2_ emulsions’ radii decreased over time for the re-emulsified system (group **A**), the higher the salinity, the greater difference in the emulsion radii. For example, the droplet size at 1,000 ppm was 17.89 μm on day 0 and day 7; however, at 25,000 ppm the droplet size was 96.22 μm on day 0 and 39.03 μm on day 7 (Figures 4 and S5). In addition, the mechanical stirring applied to regenerate the MgCl_2_ emulsion may have resulted in droplet redispersion and reorientation of the surfactant **H** leading to smaller droplets. Note that these systems were less stable and phase separation was observed 15 min after re-emulsification. In this case, less surfactant concentration (0.5% v/v) results in fewer surfactant molecules adsorbed at the oil/water interface (that provides a barrier to the coalescence of the dispersed phase); which over time resulted in less stable emulsions.^6^ Thus, droplet size measurement took place immediately after redispersion.

Group **B** emulsions presented slightly smaller droplets (see Figures 5 and S6) than group **A** systems, which could be attributed to the reduction in interfacial tension caused by higher surfactant concentration, resulting in the formation of smaller droplets. Thus, the number of droplets contacting the continuous phase increases, which ultimately increases the emulsion viscosity.^6,15^ We note that, for 5.0% v/v of **H**, salinity plays a significant role in droplet size; for example, at 8,000 ppm droplet size reached the lowest value for all the emulsions in group **B**, coinciding with the inversion of CaCl_2_ emulsions, and the multiple system (O/W/O) observed for the MgCl_2_ (Table 2 and Figure 5), yet as salinity increased, droplet size of Ca^2+^ and Mg^2+^ emulsions increased. This trend remains over time; however, for CaCl_2_ at 25,000 ppm, the radius decreased at day 7 (39.78 μm); see Figure 5. Although emulsion aging is generally expected to result in an increase in droplet size due to coalescence (or Ostwald ripening), a decrease in the average droplet diameter measured by optical microscopy is likely attributable to the sampling protocol rather than to an actual reduction in droplet size. The 25,000 ppm of CaCl_2_ emulsion is a W/O system, in which the dispersed phase (Ca^2+^ brine) has a higher density than oil. Consequently, during aging, the larger water droplets tend to sediment more rapidly toward the bottom of the vial. Since samples for microscopic analysis were collected from near the top of the glass bottle, the observed decrease in average droplet diameter most likely reflects a sampling bias associated with the sampling location and gravitational separation of the emulsion, rather than a true decrease in droplet size.

**Figure 5:**
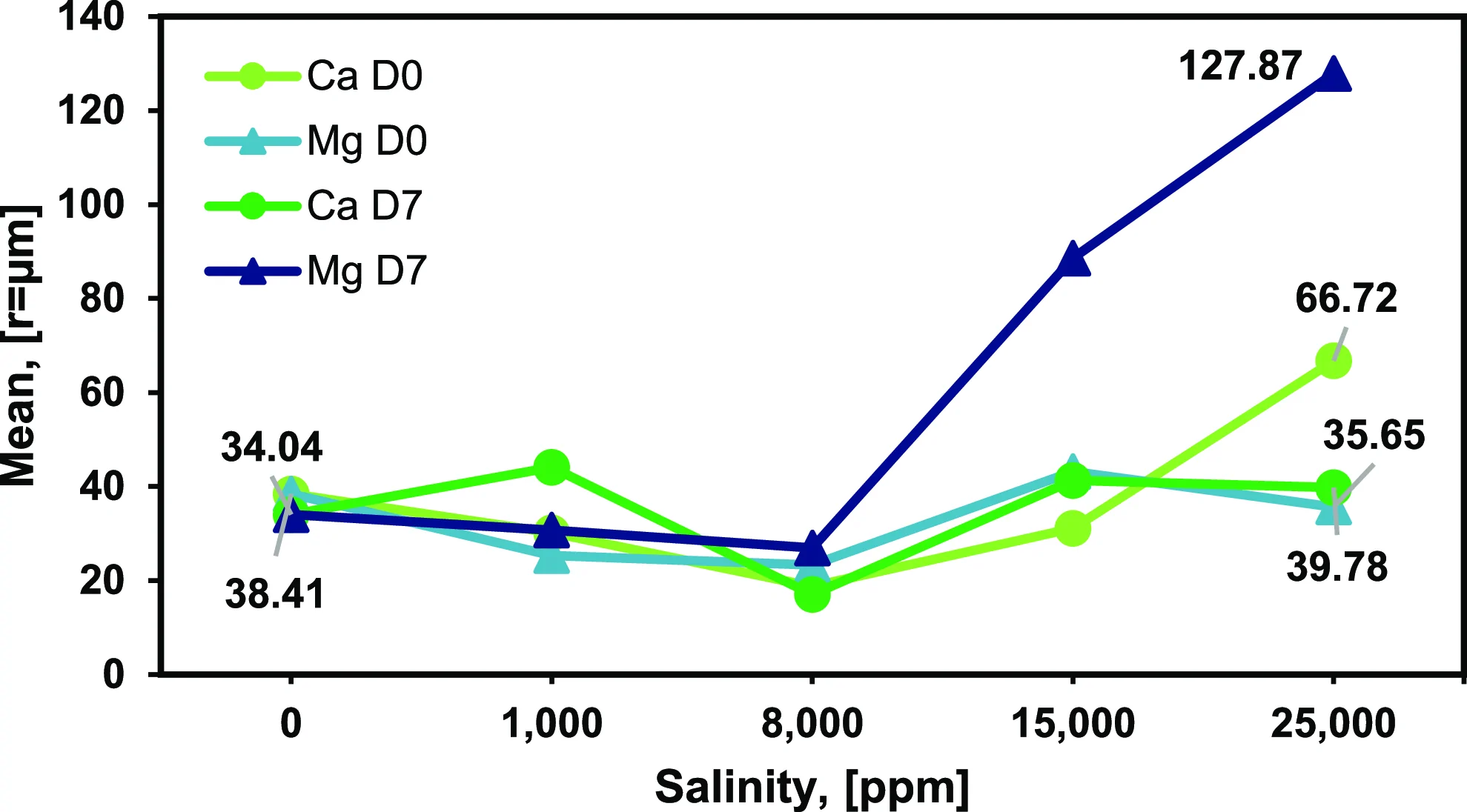
Mean radius vs salinity for 5% v/v surfactant concentration over time. The droplets’ size of MgCl_2_ emulsions increased considerably over time (day 7) compared to CaCl_2_ emulsions.

At 25,000 ppm, the interplay between droplet size, viscosity, and stability becomes most evident when comparing the two divalent cations. CaCl_2_ emulsions (group **B**) retained relatively small droplet radii (∼39.78 μm, at day 7) together with the highest viscosity values recorded across all systems, consistent with strong electrostatic bridging between Ca^2+^ and the carboxylate headgroups, which promotes droplet flocculation, a rigid interfacial film, and restricted droplet mobility under shear. In contrast, MgCl_2_ emulsions (**B**) at 25,000 ppm exhibited substantially larger droplet radii (127.87 μm, at day 7) and lower viscosity, reflecting weaker ion-headgroup interactions due to the strongly hydrated nature of Mg^2+^, which limits electrostatic bridging and interfacial film rigidity. As a result, MgCl_2_ droplets coalesce more readily over time, producing larger, more polydisperse droplets and a mechanically weaker emulsion. These results indicate that at high salinity, droplet size and viscosity are not independent outcomes of ionic strength alone but are jointly governed by the specific ion-surfactant interaction: stronger electrostatic bridging (Ca^2+^) sustains smaller droplets, higher viscosity, and greater stability, whereas weaker bridging (Mg^2+^) results in larger droplets, lower viscosity, and reduced stability. This contrast reinforces that ionic specificity, rather than salinity per se, is the dominant factor controlling emulsion stability at the highest salinities tested.

In the case of MgCl_2_ emulsions (W/O) at 15,000 and 25,000 ppm, the droplet radii increased with salinity, going from 34.04 μm (0 ppm) to 127.87 μm (25,000 ppm) at day 7 (Figure 5). Because Mg^2+^ systems were consistently less stable than the Ca^2+^ systems, coalescence occurs faster, leading to larger water droplets and lower viscosity (Figures 3 and S3).

Figures S5 and S6 show histograms generated using ROJA (our Python-based image processing tool, see Supporting Information). Statistical droplet size data are provided in Table S7; the distribution asymmetry is attributed to the stability of the systems, which in general decreases with increasing salinity, resulting in higher droplet size and polydispersity that increase hydrodynamic interactions, leading to non-Newtonian behavior. Overall, MgCl_2_ systems exhibited lower viscosity than CaCl_2_ systems (and higher polydispersity), see Figures 5 and S6. This difference was greater at higher salinities for group B. This trend likely results from a combined effect of ion-specific interactions with the surfactant headgroups and the consequent interfacial hydration/structuring, which alter emulsion stability and the ability of droplets to rearrange under shear. Consequently, the dominant mechanisms controlling viscosity may vary with the interfacial structure and the emulsion response to applied shear rather than being explained by a single route.

In Figure 6 we show the relationship between measured shear stress and droplet size for CaCl_2_ and MgCl_2_ emulsion (group **A**, day 0). For MgCl_2_, higher measured shear stress is associated with larger mean droplet size, whereas for CaCl_2_, no clear trend was observed. Notably, shear stress is a response variable rather than an independently controlled processing parameter; which depends on the emulsion’s rheology and microstructure. Therefore, the observed correlation does not allow us to unambiguously discriminate whether larger droplets arise due to shear-induced coalescence or whether changes in droplet size/microstructure modify the rheological response and, thus, the measured shear stress. Nevertheless, we observed that magnesium-containing emulsions exhibited lower viscosity than calcium emulsions, while also showing larger mean droplet sizes. Therefore, these findings suggest that ionic composition alters both the rheological response and the emulsion stability, leading to coupled rheology/microstructure behavior. Overall, the data indicate that ionic composition impacts emulsion stability and the coupled rheology/microstructure response through multiple, nonexclusive pathways.

**Figure 6:**
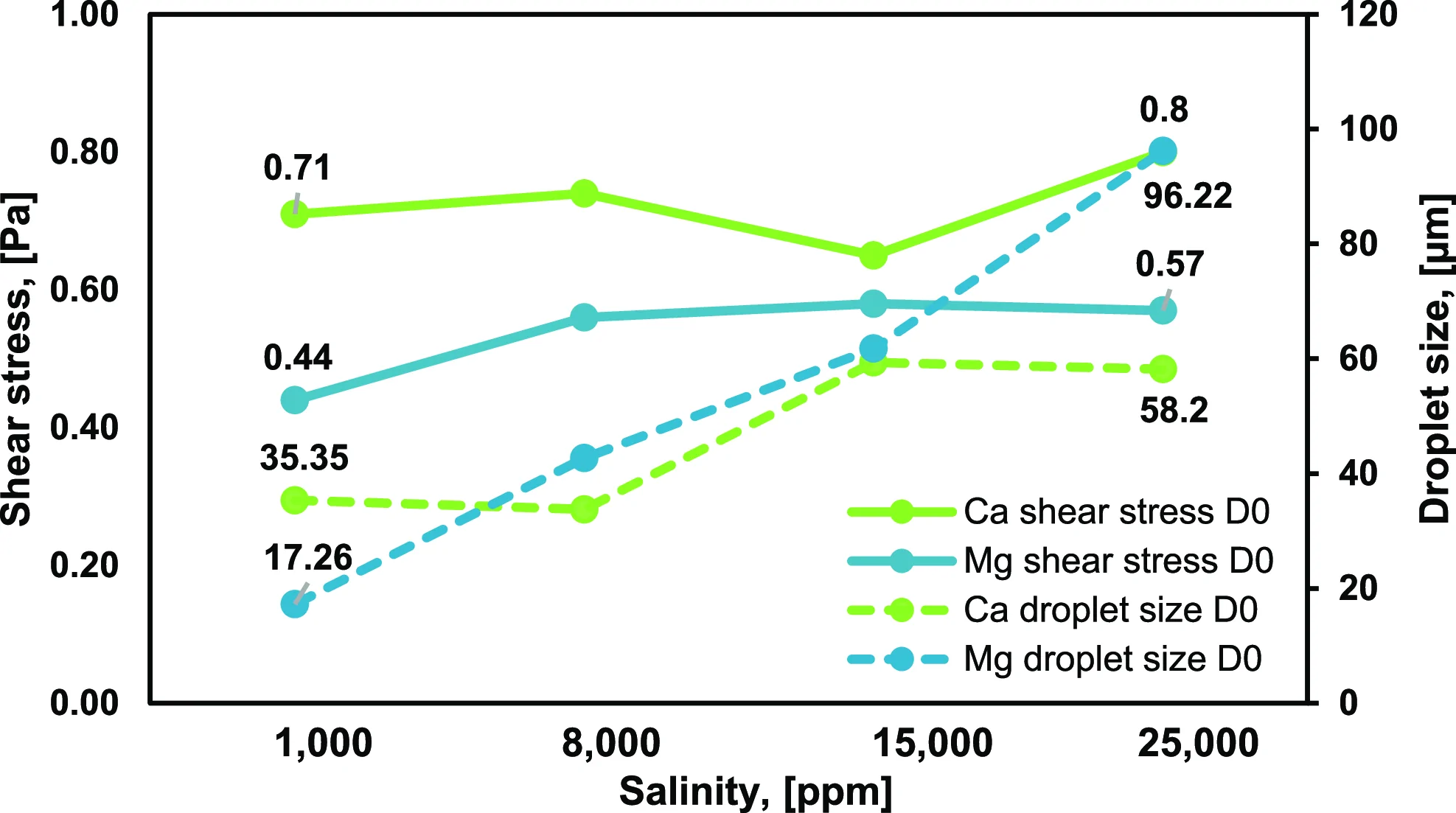
Mean radius and droplet size at different salinities for 0.5% v/v surfactant concentration, under a constant shear rate of 4.17 s^–1^. The droplet size of MgCl_2_ emulsions increased as the shear stress increased.

## 8 Conclusions

This study investigated the effects of divalent ions, calcium (Ca^2+^) and magnesium (Mg^2+^), on the emulsification performance over time, using an anionic surfactant **H** at two concentrations (0.5% and 5.0% v/v). The surfactant **H** formed stable macroemulsions in the presence of divalent ions but produced unstable emulsions with sodium ions (Na^+^), confirming that ionic valence plays a decisive role in emulsion stability.

At low surfactant concentration (0.5% v/v), droplet size in MgCl_2_ emulsions increased with salinity (from 17.89 μm at 1,000 ppm to 96.22 μm at 25,000 ppm on day 0), resulting in less stable systems compared to CaCl_2_ emulsions. Viscosity in CaCl_2_ increased with time and salinity, reaching up to 240.00 cP at 25,000 ppm (day 7, 5.0% v/v), representing up to one order of magnitude higher viscosity than equivalent MgCl_2_ systems. In contrast, MgCl_2_ emulsions did not show a significant increase in viscosity over time, which is consistent with the larger droplet sizes and lower droplet–droplet interaction observed for this system.

At higher surfactant concentration (5.0% v/v), both CaCl_2_ and MgCl_2_ emulsions showed phase inversion phenomena; at 8,000 ppm CaCl_2_ inverted to W/O systems, while MgCl_2_ emulsions formed multiple (O/W/O) systems coinciding with the minimum droplet size observed across all the emulsions in group B.

Overall, CaCl_2_ emulsions exhibited higher viscosities than MgCl_2_ systems across all of the conditions tested, particularly at high salinity and shear rate (4.17 s^–1^). This behavior is likely due to stronger electrostatic interactions between Ca^2+^ and the carboxylate headgroups of the anionic surfactant, promoting droplet flocculation, rigid interfacial films, and an increased apparent viscosity. In comparison, Mg^2+^ ions retain a stronger hydration shell, limiting their interaction with the surfactant headgroups and resulting in larger droplets, weaker droplet–droplet interactions, and a lower viscosity.

Emulsion studies often produce large datasets that require specialized tools for efficient processing and interpretation. To address this need, we developed ROJA, a flexible tool that integrates advanced image-processing algorithms and customizable filtering techniques to ensure accurate, reproducible droplet analysis.

## Supplementary Material



## Notes

Series of supplementary figures and tables providing detailed experimental data on the physical properties of various oil–water emulsions, including surface tension, droplet size distribution, shear stress, and rheological behavior (fluency index) under varying conditions of salinity, with CaCl_2_ and MgCl_2_ brines, surfactant concentration, and time. Furthermore, the document introduces and describes ROJA, a custom Python-based image processing program developed to accurately segment, measure, and statistically analyze emulsion droplets, overcoming the limitations of standard software like ImageJ in noisy imaging environments. The surfactant characterization is also included (PDF)

## Notes

Declaration of AI tool technologies used in the manuscript preparation process. The authors used Claude Sonnet 5 to revise grammar, language, and the manuscript’s proofreading, and to highlight important aspects, maintain original ideas, and focus on the study’s key findings, while organizing them in a more structured and cohesive manner. After using this tool, the authors reviewed and edited the content as needed and take full responsibility for the content of the manuscript.
