# Supplementary material for: Rheological Insights: The Role of Divalent Ions on the Stability and Phase Behavior of Oil–Water Emulsions
Source: ACS Omega. 2026 Sep 29;11(40):60713–21. doi: 10.1021/acsomega.6c01366 (PMC13670862; doi:10.1021/acsomega.6c01366)
Supplement: Supplementary file 1 [file ao6c01366_si_001.pdf]

Supporting Information for:

Rheological Insights: The Role of Divalent Ions on the Stability and Phase Behavior of Oil-Water Emulsions

Janett Montaño-Salazar[a], A. Paulina Gómora-Figueroa[a], Roberto Zenit[b], Rosa María Mariscal-Romero[a]*

[a] División de Ingeniería en Ciencias de la Tierra, Facultad de Ingeniería, Universidad Nacional Autónoma de México, Circuito Exterior, Ciudad Universitaria, 04510, Ciudad de México, Mexico

[b] School of Engineering, Brown University, 184 Hope St, Providence, Rhode Island 02912, USA

***Corresponding Author:** p.gomora@unam.mx

TABLE OF CONTENTS

FIGURES

Figure S1. Surface tension of $CaCl_2$ and $MgCl_2$ brines (0 – 25,000 ppm) and surfactant (H) concentration of 0.5% v/v.

Figure S2. Surface tension of $CaCl_2$ and $MgCl_2$ brines (0 – 25,000 ppm) and surfactant (H) concentration of 5% v/v.

Figure S3. Emulsions formed with 0.5% v/v of H (A) and 5% v/v (B) on day 7.

Figure S4. $CaCl_2$ systems from group A at day 7 and day 20.

Figure S5. Droplet size distribution for $MgCl_2$ and $CaCl_2$ emulsions at days 0 (left) and 7 (right), using 0.5% v/v of surfactant concentration. Salinity ranges from 0 to 25,000 ppm.

Figure S6. Droplet size distribution for $MgCl_2$ and $CaCl_2$ emulsions at days 0 (left) and 7 (right), using 5% v/v of surfactant concentration. Salinity ranges from 0 to 25,000 ppm.

Figure S7. (A) shows the visualization of the original image. (B) presents the grayscale image processed with a Gaussian smoothing filter from skimage to reduce noise and improve segmentation. (C) displays the initial object mask and thresholding method (skimage.filters.threshold) to create a binary image where objects of interest are foreground (colors) and the background is black. Statistical data, including noise, are also generated for the objects.

Figure S8. Heatmap showing droplet area distribution map in an emulsion, where each color represents a range of droplet areas in mm^2

Figure S9. Shear stress and droplet size for emulsions prepared with $CaCl_2$ and $MgCl_2$ brines, surfactant (H) at concentrations of 0.5%v/v on day 0, with a shear rate of 4.17 s^{-1}.

Figure S10. Shear stress and droplet size for emulsions prepared with $CaCl_2$ and $MgCl_2$ brines, surfactant (H) at concentrations of 5.0%v/v on day 0, with a shear rate of 4.17 s^{-1}.

Figure S11. Anionic surfactant (H) Infrared Spectra

Figure S12. Anionic surfactant (H) ^{1}H NMR

Figure S13. Anionic surfactant (H) ^{13}C NMR

TABLES

Table S1. Type of emulsion, fluency index "n", and rheological behavior with salinity for $CaCl_2$ and $MgCl_2$ at 0.5% v/v of surfactant on day 7.

Table S2. Type of emulsion, fluency index "n", and rheological behavior with salinity for $CaCl_2$ and $MgCl_2$ at 5.0% v/v of surfactant on day 7.

Table S3. Shear stress for emulsions prepared with $CaCl_2$ brines and surfactant (H) concentration of 0.5%v/v and 5%v/v on day 0.

Table S4. Shear stress for emulsions prepared with $MgCl_2$ brines and surfactant (H) concentration of 0.5%v/v and 5%v/v on day 0.

Table S5. Shear stress for emulsions prepared with $CaCl_2$ brines and surfactant (H) concentration of 0.5%v/v and 5%v/v on day 7.

Table S6. Shear stress for emulsions prepared with $MgCl_2$ brines and surfactant (H) concentration of 0.5%v/v and 5%v/v on day 7.

Table S7. Statistic numbers of analyzed droplets in Excel format with ROJA_LIRFFF.

Table S8. Shear stress and droplet size for emulsions prepared with $CaCl_2$ brines and surfactant (H) at concentrations of 0.5%v/v and 5%v/v on day 0, with a shear rate of 4.17 s^{-1}.

Table S9. Shear stress and droplet size for emulsions prepared with $MgCl_2$ brines and surfactant (H) at concentrations of 0.5%v/v and 5%v/v on day 0, with a shear rate of 4.17 s^{-1}.

Table S10. Shear stress and droplet size for emulsions prepared with $CaCl_2$ brines and surfactant (H) at concentrations of 0.5%v/v and 5%v/v on day 7, with a shear rate of 4.17 s^{-1}.

Table S11. Shear stress and droplet size for emulsions prepared with $MgCl_2$ brines and surfactant (H) at concentrations of 0.5%v/v and 5%v/v on day 7, with a shear rate of 4.17 s^{-1}.

ROJA

Program description

CODE

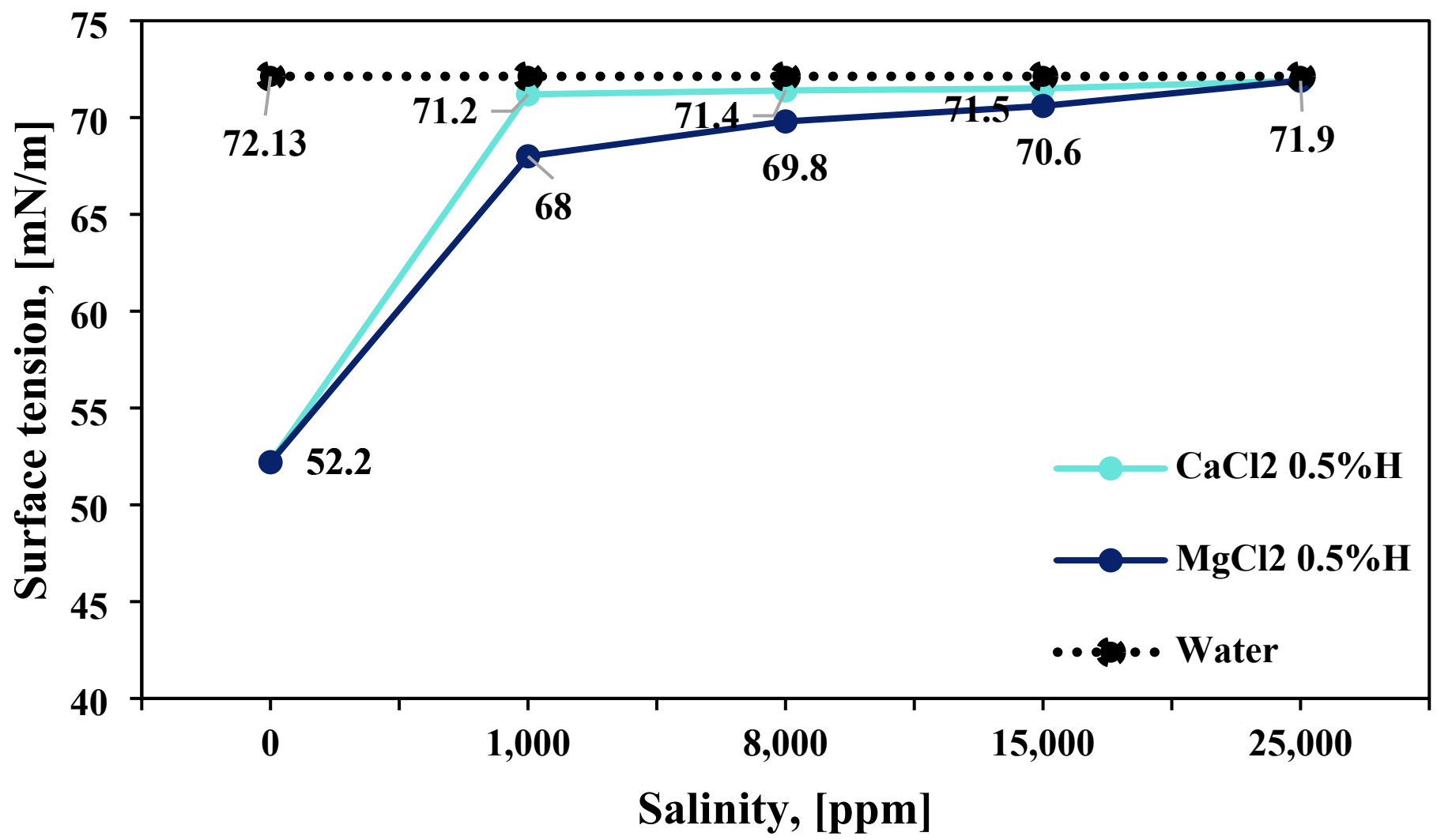

Figure S1. Surface tension of $CaCl_2$ and $MgCl_2$ brines (0 – 25,000 ppm) and surfactant (H) concentration of 0.5% v/v.

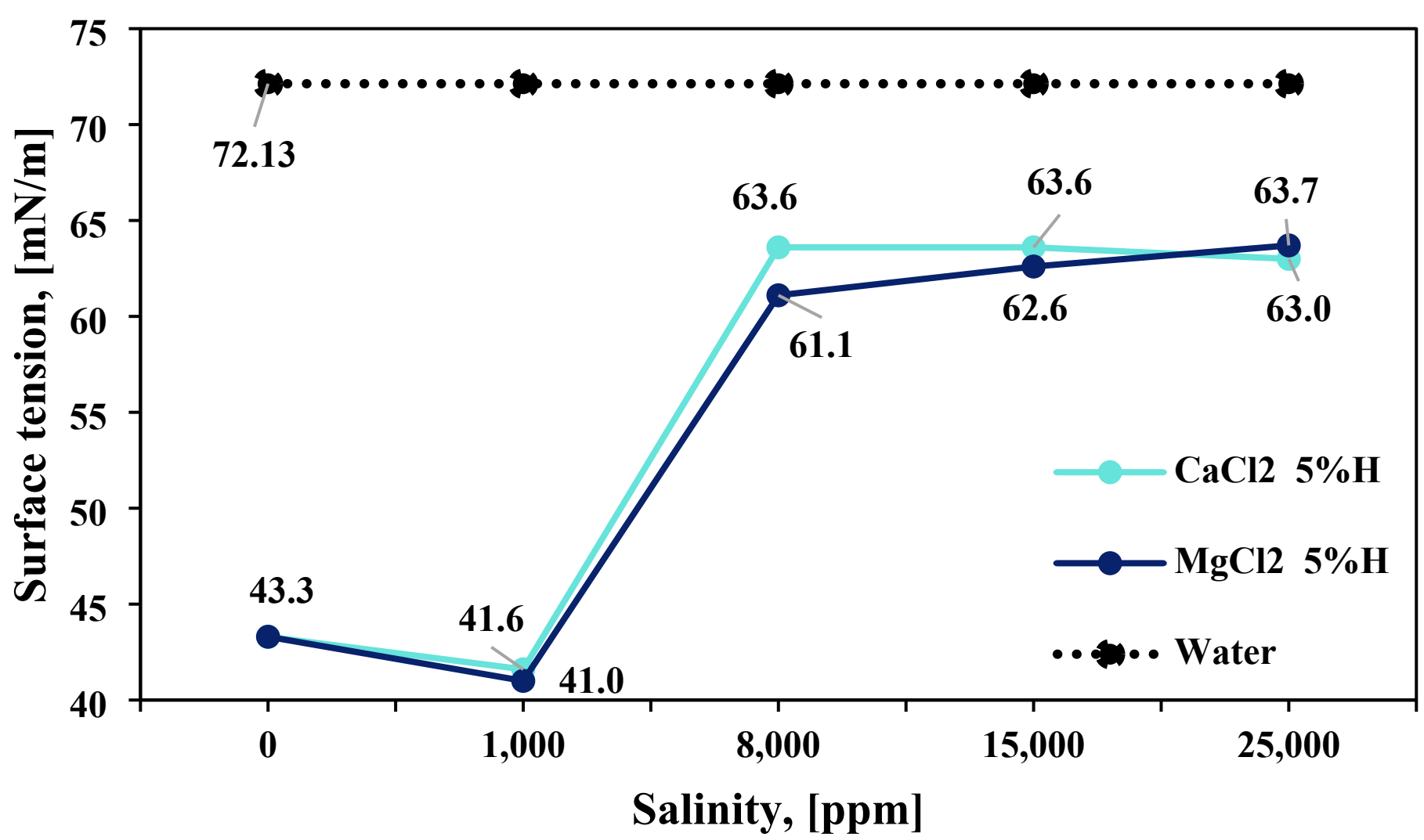

Figure S2. Surface tension of $CaCl_2$ and $MgCl_2$ brines (0 – 25,000 ppm) and surfactant (H) concentration of 5% v/v.

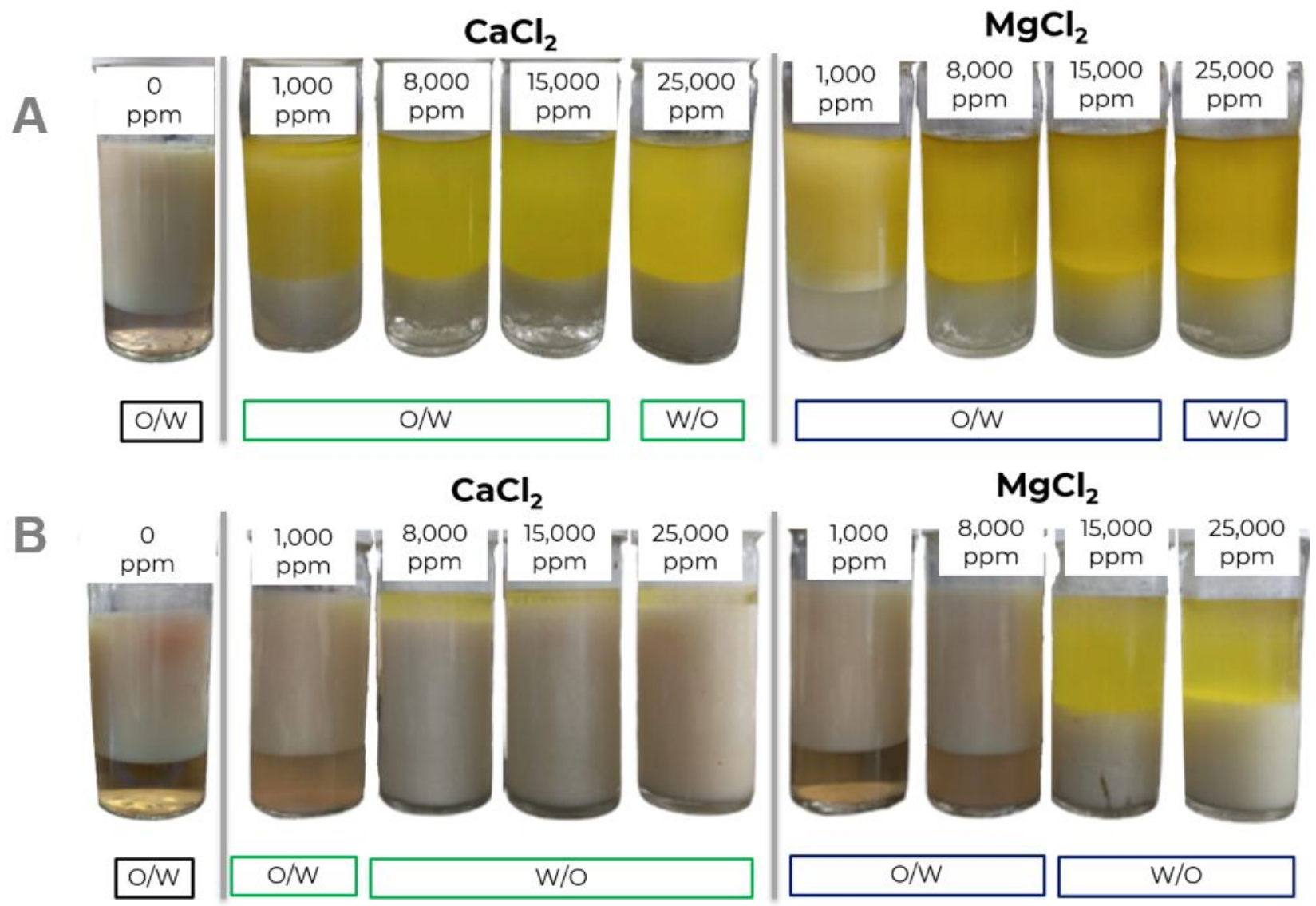

Figure S3. Emulsions formed with 0.5% v/v of H (A) and 5% v/v (B) on day 20, after characterization.

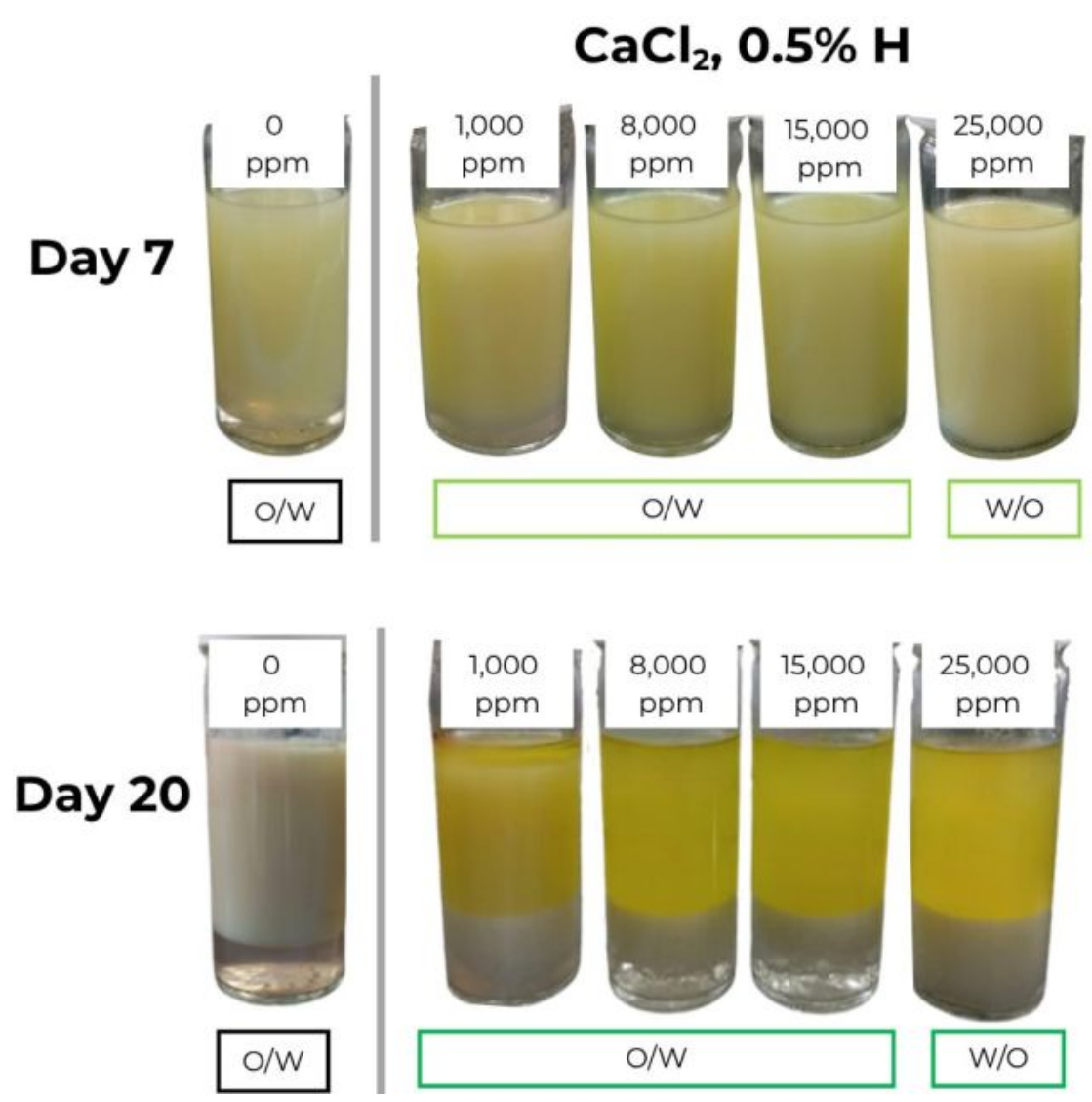

Figure S4. $CaCl_2$ systems from group A at day 7 and day 20.

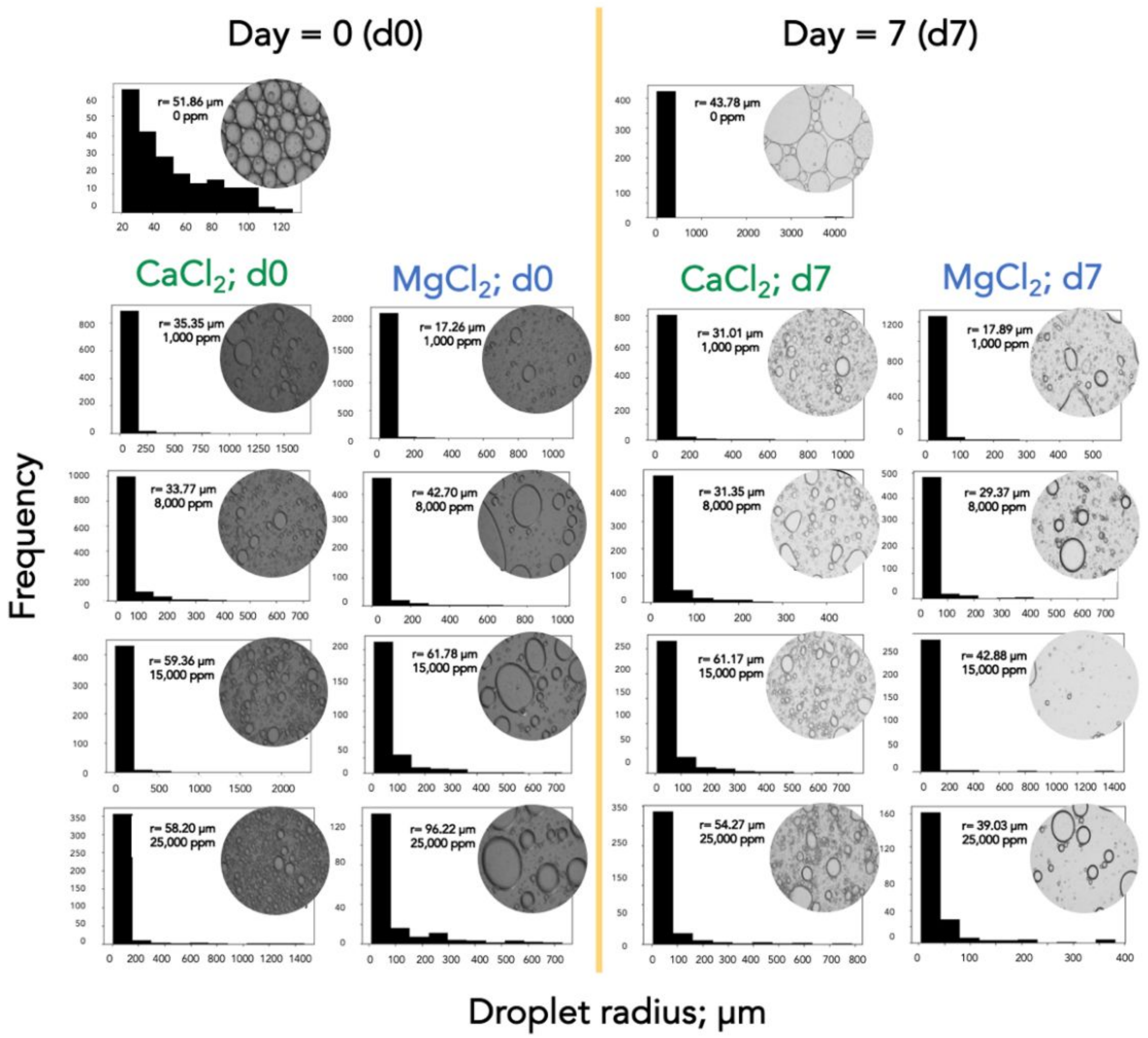

Figure S5. Droplet size distribution for $MgCl_2$ and $CaCl_2$ emulsions at days 0 (left) and 7 (right), using 0.5% v/v of surfactant concentration. Salinity ranges from 0 to 25,000 ppm.

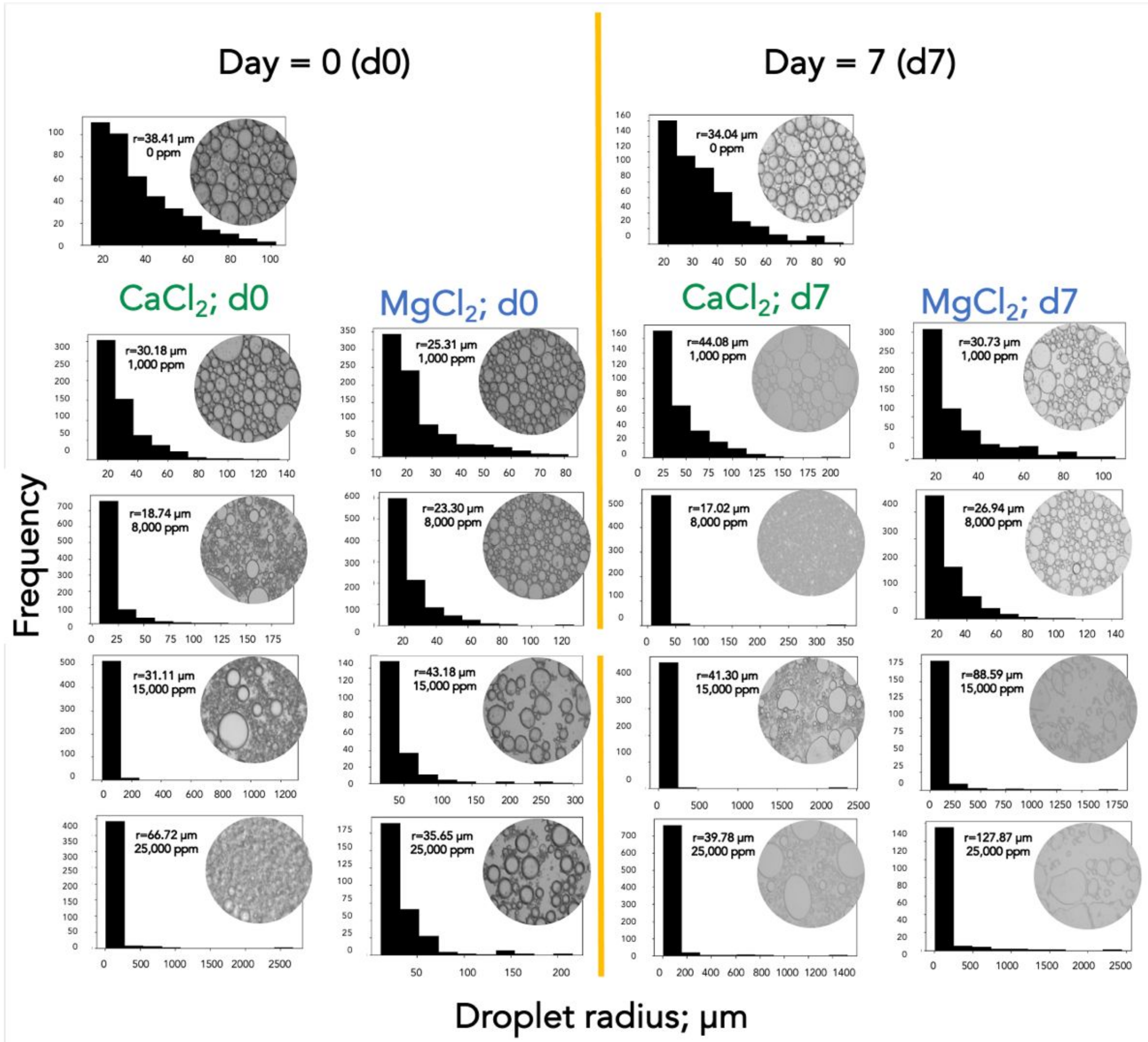

Figure S6. Droplet size distribution for $MgCl_2$ and $CaCl_2$ emulsions at days 0 (left) and 7 (right), using 5% v/v of surfactant concentration. Salinity ranges from 0 to 25,000 ppm.

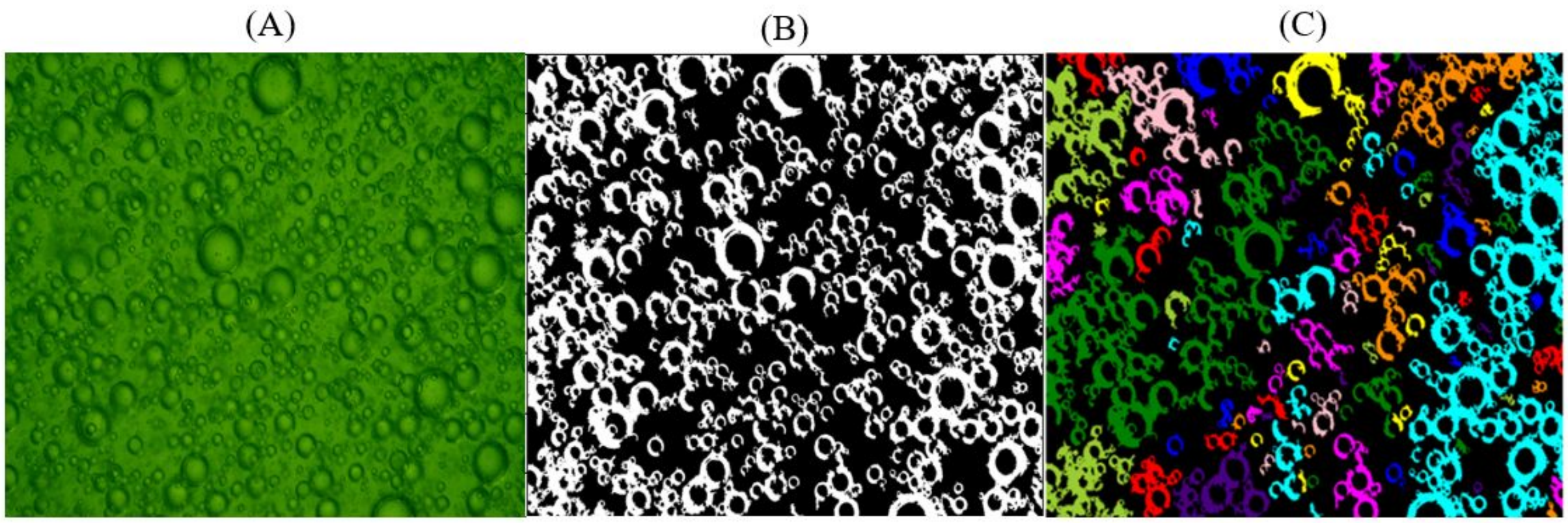

Figure S7. (A) shows the visualization of the original image. (B) presents the grayscale image processed with a Gaussian smoothing filter from skimage to reduce noise and improve segmentation. (C) displays the initial object mask and thresholding method (skimage.filters.threshold) to create a binary image where objects of interest are foreground (colors) and the background is black. Statistical data, including noise, are also generated for the objects.[1]

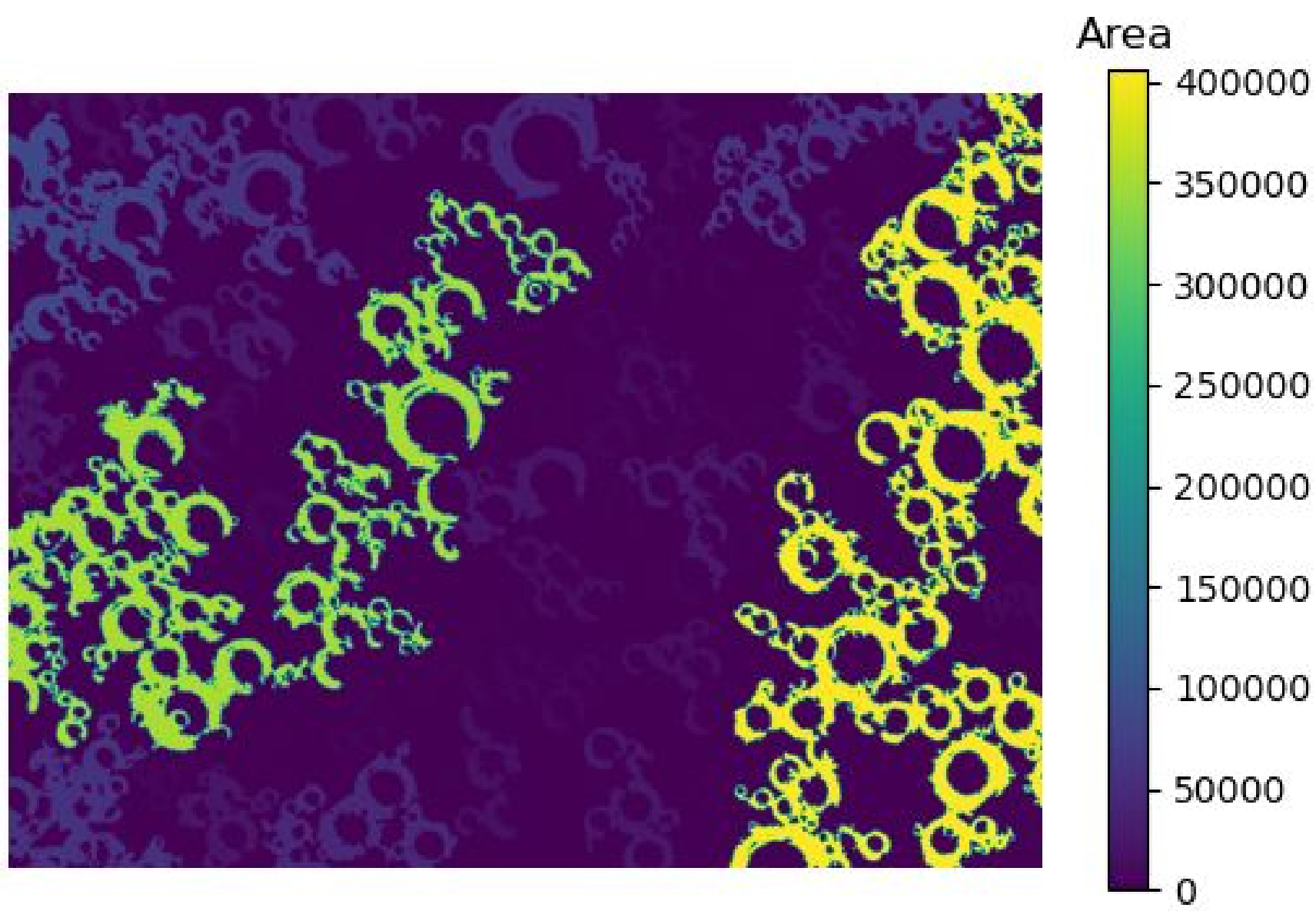

Figure S8. Heatmap showing droplet area distribution in an emulsion, where each color represents a range of droplet areas in mm^2.

[1] J. Montaño-Salazar, R. M. Mariscal-Romero, A. P. Gómora-Figueroa, A. Peralta-Jarquín A cuentagotas: Un vistazo a la distribución del tamaño de gotas y su influencia en las propiedades de emulsiones. Revista Materiales Avanzados, vol 41, 25 – 38, 2024. https://revista.iim.unam.mx/index.php/materialesa_avanzados/article/view/98/87

Table S1. Type of emulsion, fluency index "n", and rheological behavior with salinity for $CaCl_2$ and $MgCl_2$ at 0.5% v/v of surfactant on day 7.

Salinity ppm	$CaCl_2$ 0.5% D7			$MgCl_2$ 0.5% D7		
	emulsion type	rheological behavior	n	emulsion type	rheological behavior	n
0	O/W	shear thinning	0.561	O/W	shear thinning	0.561
1,000	O/W	shear thinning	0.986	O/W	shear thinning	0.945
8,000	O/W	shear thickening	1.069	O/W	shear thinning	0.915
15,000	O/W	shear thickening	1.02	O/W	shear thinning	0.927
25,000	W/O	shear thinning	0.98	W/O	shear thinning	0.939

Table S2. Type of emulsion, fluency index "n", and rheological behavior with salinity for $CaCl_2$ and $MgCl_2$ at 5.0% v/v of surfactant on day 7.

Salinity ppm	$CaCl_2$ 5% D7			$MgCl_2$ 5% D7		
	emulsion type	rheological behavior	n	emulsion type	rheological behavior	n
0	O/W	shear thinning	0.709	O/W	shear thinning	0.709
1,000	O/W	shear thinning	0.754	O/W	shear thinning	0.731
8,000	W/O	shear thinning	0.828	O/W	shear thinning	0.646
15,000	W/O	shear thinning	0.723	W/O	shear thinning	0.691
25,000	W/O	shear thinning	0.726	W/O	shear thinning	0.818

Table S3. Shear stress for emulsions prepared with $CaCl_2$ brines and surfactant **H** concentration of 0.5%v/v and 5%v/v on day 0.

		$CaCl_2$ (H 0.5% v/v) Shear stress [Pa]					$CaCl_2$ (H 5% v/v) Shear stress [Pa]				
Shear rate [s^{-1}]	Oil	0 ppm	1,000 ppm	8,000 ppm	15,000 ppm	25,000 ppm	0 ppm	1,000 ppm	8,000 ppm	15,000 ppm	25,000 ppm
**0.5**	0.03	0.07	0.08	0.08	0.07	0.1	0.04	0.05	0.12	0.15	0.14
**1**	0.06	0.1	0.16	0.18	0.15	0.2	0.05	0.08	0.22	0.26	0.25
**2**	0.13	0.15	0.33	0.36	0.32	0.39	0.09	0.12	0.39	0.44	0.43
**4**	0.29	0.23	0.68	0.71	0.62	0.77	0.16	0.19	0.72	0.77	0.77
**4.17**	0.3	0.24	0.71	0.74	0.65	0.8	0.17	0.2	0.75	0.79	0.79

Table S4. Shear stress for emulsions prepared with $MgCl_2$ brines and surfactant **H** concentration of 0.5%v/v and 5%v/v on day 0.

		$MgCl_2$ (H 0.5% v/v) Shear stress [Pa]					$MgCl_2$ (H 5% v/v) Shear stress [Pa]				
**Shear rate [s^{-1}]**	**Oil**	**0 ppm**	**1,000 ppm**	**8,000 ppm**	**15,000 ppm**	**25,000 ppm**	**0 ppm**	**1,000 ppm**	**8,000 ppm**	**15,000 ppm**	**25,000 ppm**
**0.5**	0.03	0.07	0.05	0.08	0.09	0.08	0.04	0.06	0.05	0.14	0.16
**1**	0.06	0.10	0.10	0.15	0.16	0.14	0.05	0.08	0.07	0.23	0.28
**2**	0.13	0.15	0.20	0.28	0.29	0.28	0.09	0.13	0.11	0.38	0.46
**4**	0.29	0.23	0.42	0.53	0.55	0.54	0.16	0.21	0.18	0.65	0.76
**4.17**	0.30	0.24	0.44	0.56	0.58	0.57	0.17	0.22	0.19	0.67	0.78

Table S5. Shear stress for emulsions prepared with $CaCl_2$ brines and surfactant **H** concentration of 0.5%v/v and 5%v/v on day 7.

		$CaCl_2$ (H 0.5% v/v) Shear stress [Pa]					$CaCl_2$ (H 5% v/v) Shear stress [Pa]				
**Shear rate [s^{-1}]**	**Oil**	**0 ppm**	**1,000 ppm**	**8,000 ppm**	**15,000 ppm**	**25,000 ppm**	**0 ppm**	**1,000 ppm**	**8,000 ppm**	**15,000 ppm**	**25,000 ppm**
**0.5**	0.03	0.07	0.08	0.08	0.07	0.10	0.04	0.15	0.19	0.21	0.21
**1**	0.06	0.10	0.16	0.18	0.15	0.20	0.06	0.24	0.31	0.35	0.35
**2**	0.13	0.15	0.33	0.36	0.32	0.39	0.10	0.40	0.53	0.56	0.57
**4**	0.29	0.23	0.68	0.71	0.62	0.77	0.19	0.70	0.93	0.95	0.97
**4.17**	0.30	0.24	0.71	0.74	0.65	0.80	0.19	0.73	1.25	0.98	1.00

Table S6. Shear stress for emulsions prepared with $MgCl_2$ brines and surfactant **H** concentration of 0.5%v/v and 5%v/v on day 7.

		$MgCl_2$ (H 0.5% v/v) Shear stress [Pa]					$MgCl_2$ (H 5% v/v) Shear stress [Pa]				
**Shear rate [s^{-1}]**	**Oil**	**0 ppm**	**1,000 ppm**	**8,000 ppm**	**15,000 ppm**	**25,000 ppm**	**0 ppm**	**1,000 ppm**	**8,000 ppm**	**15,000 ppm**	**25,000 ppm**
**0.5**	0.03	0.07	0.05	0.08	0.09	0.08	0.04	0.04	0.05	0.16	0.11
**1**	0.06	0.10	0.10	0.15	0.16	0.14	0.06	0.07	0.07	0.26	0.21
**2**	0.13	0.15	0.20	0.28	0.29	0.28	0.10	0.11	0.11	0.42	0.37
**4**	0.29	0.23	0.42	0.53	0.55	0.54	0.19	0.19	0.18	0.68	0.63
**4.17**	0.30	0.24	0.44	0.56	0.58	0.57	0.19	0.20	0.19	0.70	0.65

Table S7. Statistic numbers of analyzed droplets in Excel format with ROJA.

GROUP A

**(control) 0.5%H Day 0**							
**0 ppm**	**got_no**	**got_min**	**got_max**	**got_mean**	**got_median**	**got_mode**	**got_std**
	218	0.0207516	0.19365048	0.05185809	0.04367874	0.02277838	0.027599
**$CaCl_2$ 0.5%H Day 0**							
	**got_no**	**got_min**	**got_max**	**got_mean**	**got_median**	**got_mode**	**got_std**
**1,000 ppm**	924	0.00472965	2.73851385	0.03534747	0.00837376	0.00585112	0.14420535
**8,000 ppm**	1127	0.00558018	0.7169892	0.03377169	0.01174902	0.00702725	0.06272831
**15,000 ppm**	448	0.00692103	4.05034508	0.059361	0.01227159	0.00723993	0.25704553
**25,000 ppm**	379	0.0075556	2.36657708	0.05819876	0.01603712	0.00912081	0.18754753
**$MgCl_2$ 0.5%H Day 0**							
	**got_no**	**got_min**	**got_max**	**got_mean**	**got_median**	**got_mode**	**got_std**
**1,000 ppm**	2278	0.00373612	1.10930881	0.01725703	0.00700448	0.00380797	0.05243451
**8,000 ppm**	501	0.00471069	2.36291947	0.04270148	0.00851637	0.00653851	0.14467451
**15,000 ppm**	268	0.00627934	0.9630518	0.0617754	0.02122836	0.25576187	0.10516647
**25,000 ppm**	180	0.00952528	1.85526764	0.09621628	0.02266384	0.00971441	0.18884685
**(control) 0.5%H Day 7**							
**0 ppm**	**got_no**	**got_min**	**got_max**	**got_mean**	**got_median**	**got_mode**	**got_std**
	170	0.00405023	3.86752553	0.04424082	0.00714313	0.00464672	0.29542702
**$CaCl_2$ 0.5%H Day 7**							
	**got_no**	**got_min**	**got_max**	**got_mean**	**got_median**	**got_mode**	**got_std**
**1,000 ppm**	467	0.00505283	1.36362748	0.03153861	0.01071734	0.00695781	0.09393573
**8,000 ppm**	391	0.00528367	0.47351542	0.0298359	0.0139769	0.00578043	0.05051646
**15,000 ppm**	215	0.00726172	1.53796804	0.05437025	0.01464484	0.00999291	0.14200449
**25,000 ppm**	224	0.00658537	1.03752479	0.05256746	0.01473768	0.00716057	0.12699513
**$MgCl_2$ 0.5%H Day 7**							
	**got_no**	**got_min**	**got_max**	**got_mean**	**got_median**	**got_mode**	**got_std**
**1,000 ppm**	460	0.00507051	1.33672078	0.02618829	0.00922129	0.00736964	0.08989409
**8,000 ppm**	346	0.00419492	0.92003488	0.02613457	0.00733503	0.00420914	0.06973695
**15,000 ppm**	194	0.00378372	1.51602525	0.04521804	0.00952236	0.00383101	0.14535682
**25,000 ppm**	93	0.00697255	0.66826153	0.07003653	0.01558491	0.00697255	0.11665211

GROUP B

**(control) 5%H Day 0**							
**0 ppm**	**got_no**	**got_min**	**got_max**	**got_mean**	**got_median**	**got_mode**	**got_std**
	410	0.01632927	0.11557693	0.03841368	0.0332666	0.05643006	0.01843197
**$CaCl_2$ 5%H Day 0**							
	**got_no**	**got_min**	**got_max**	**got_mean**	**got_median**	**got_mode**	**got_std**
**1,000 ppm**	591	0.01304102	0.17209711	0.03017566	0.02497803	0.01644576	0.01829942
**8,000 ppm**	913	0.00704913	0.21625526	0.01874389	0.01227523	0.00930351	0.01925273
**15,000 ppm**	529	0.00677733	1.42549633	0.03110539	0.01437896	0.00753868	0.08844223
**25,000 ppm**	409	0.00793303	2.98421224	0.0667233	0.02082515	0.02279248	0.21889171

	got_no	got_min	got_max	got_mean	got_median	got_mode	got_std
**$MgCl_2$ 5%H Day 0**							
	**got_no**	**got_min**	**got_max**	**got_mean**	**got_median**	**got_mode**	**got_std**
**1,000 ppm**	862	0.01133274	0.09036968	0.02530546	0.02028037	0.01388939	0.01423155
**8,000 ppm**	981	0.00962186	0.15392066	0.02330168	0.01802568	0.02323086	0.01526243
**15,000 ppm**	207	0.01467258	0.30657784	0.04318063	0.03025101	0.21001223	0.04391174
**25,000 ppm**	300	0.01147398	0.23109657	0.03565376	0.0270134	0.01741812	0.03155958
**(control) 5%H Day 7**							
**0 ppm**	**got_no**	**got_min**	**got_max**	**got_mean**	**got_median**	**got_mode**	**got_std**
	215	0.01653078	0.10019472	0.03785531	0.03315164	0.02607061	0.01717902
**$CaCl_2$ 5%H Day 7**							
	**got_no**	**got_min**	**got_max**	**got_mean**	**got_median**	**got_mode**	**got_std**
**1,000 ppm**	264	0.0130456	0.13007154	0.03338978	0.02655674	0.03717234	0.02062671
**8,000 ppm**	335	0.00854135	0.35676029	0.01892855	0.01355025	0.01042813	0.02451475
**15,000 ppm**	328	0.00599752	3.05169855	0.03271014	0.0115323	0.00795691	0.17664417
**25,000 ppm**	389	0.00580033	0.84437468	0.0394904	0.01178606	0.02249055	0.09983193
**$MgCl_2$ 5%H Day 7**							
	**got_no**	**got_min**	**got_max**	**got_mean**	**got_median**	**got_mode**	**got_std**
**1,000 ppm**	365	0.01206941	0.10763108	0.02725338	0.02025556	0.02651641	0.01711201
**8,000 ppm**	392	0.01137478	0.14129475	0.02652952	0.0201537	0.01323625	0.01728143
**15,000 ppm**	99	0.0072535	1.5432923	0.0731221	0.02842094	0.21003566	0.19721154
**25,000 ppm**	133	0.00644344	1.29789464	0.05871689	0.02300638	0.14274614	0.15667541

Table S8. Shear stress and droplet size for emulsions prepared with $CaCl_2$ brines and surfactant **H**, at concentrations of 0.5%v/v and 5%v/v on day 0, with a shear rate of 4.17 s^{-1}.

	**$CaCl_2$ (H 0.5% v/v)**					**$CaCl_2$ (H 5% v/v)**				
	**0 ppm**	**1,000 ppm**	**8,000 ppm**	**15,000 ppm**	**25,000 ppm**	**0 ppm**	**1,000 ppm**	**8,000 ppm**	**15,000 ppm**	**25,000 ppm**
**Shear stress [Pa]**	0.24	0.71	0.74	0.65	0.8	0.17	0.2	0.75	0.79	0.79
**Droplet size [μm]**	51.86	35.35	33.77	59.36	58.2	38.41	30.18	18.74	31.11	66.72

Table S9. Shear stress and droplet size for emulsions prepared with $MgCl_2$ brines and surfactant **H**, at concentrations of 0.5%v/v and 5%v/v on day 0, with a shear rate of 4.17 s^{-1}.

	$MgCl_2$ (H 0.5% v/v)					$MgCl_2$ (H 5% v/v)				
	0 ppm	1,000 ppm	8,000 ppm	15,000 ppm	25,000 ppm	0 ppm	1,000 ppm	8,000 ppm	15,000 ppm	25,000 ppm
Shear stress [Pa]	0.24	0.44	0.56	0.58	0.57	0.17	0.22	0.19	0.67	0.78
Droplet size [μm]	51.86	17.26	42.7	61.78	96.22	38.41	25.31	23.3	43.18	35.65

Table S10. Shear stress and droplet size for emulsions prepared with $CaCl_2$ brines and surfactant **H**, at concentrations of 0.5%v/v and 5%v/v on day 7, with a shear rate of 4.17 s^{-1}.

	$CaCl_2$ (H 0.5% v/v)					$CaCl_2$ (H 5% v/v)				
	0 ppm	1,000 ppm	8,000 ppm	15,000 ppm	25,000 ppm	0 ppm	1,000 ppm	8,000 ppm	15,000 ppm	25,000 ppm
Shear stress [Pa]	0.24	0.71	0.74	0.65	0.8	0.19	0.73	1.25	0.98	1
Droplet size [μm]	43.78	31.01	31.35	61.17	54.27	34.04	44.08	17.02	41.3	39.78

Table S11. Shear stress and droplet size for emulsions prepared with $MgCl_2$ brines and surfactant **H**, at concentrations of 0.5%v/v and 5%v/v on day 7, with a shear rate of 4.17 s^{-1}.

	$MgCl_2$ (H 0.5% v/v)					$MgCl_2$ (H 5% v/v)				
	0 ppm	1,000 ppm	8,000 ppm	15,000 ppm	25,000 ppm	0 ppm	1,000 ppm	8,000 ppm	15,000 ppm	25,000 ppm
Shear stress [Pa]	0.24	0.44	0.56	0.58	0.57	0.19	0.2	0.19	0.7	0.65
Droplet size [μm]	43.78	17.89	29.37	42.88	39.03	34.04	30.73	26.94	88.59	127.87

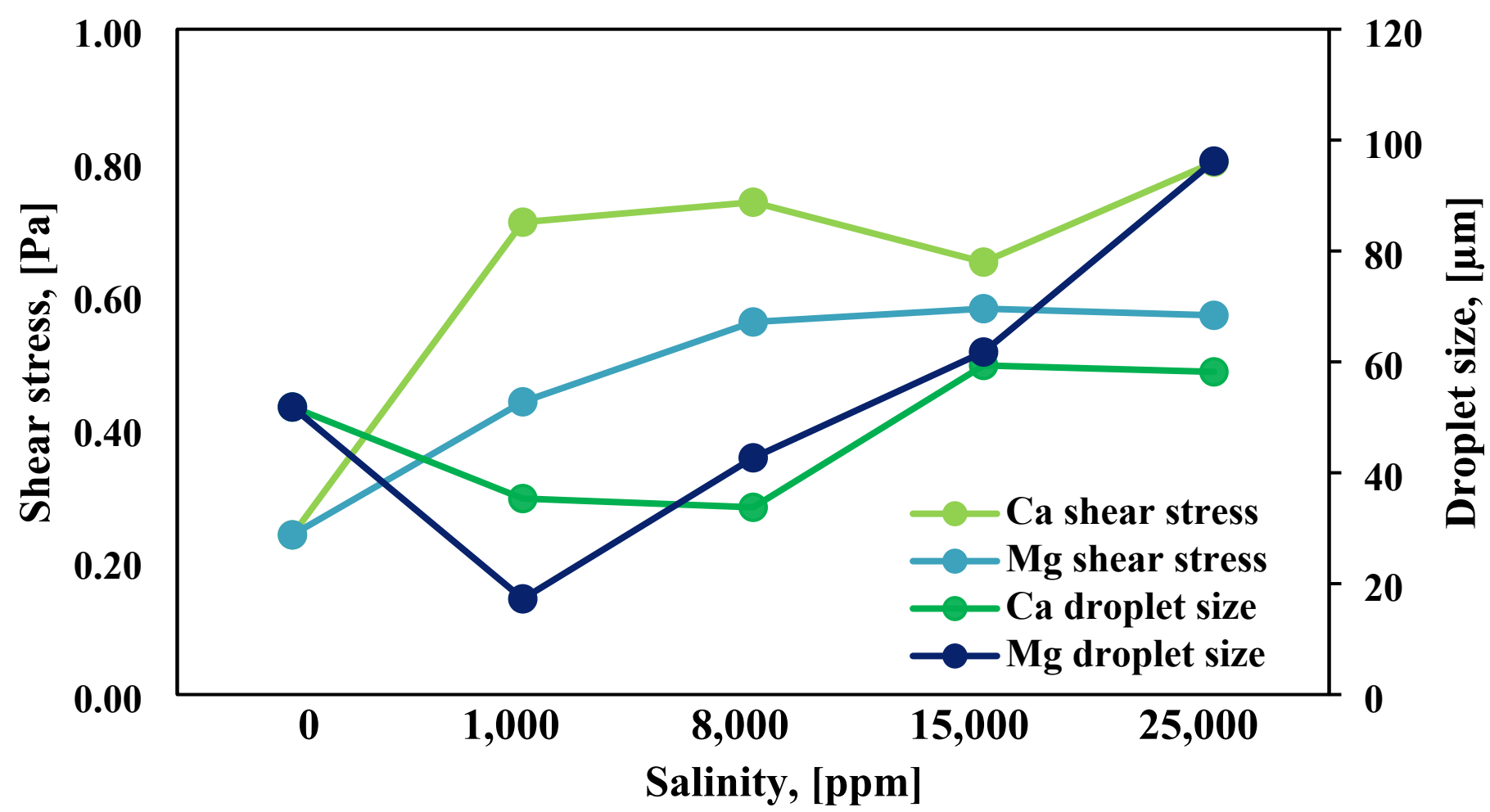

Figure S9. Shear stress and droplet size for emulsions prepared with $CaCl_2$ and $MgCl_2$ brines, surfactant (H) at concentrations of 0.5%v/v on day 0, with a shear rate of 4.17 s^{-1}.

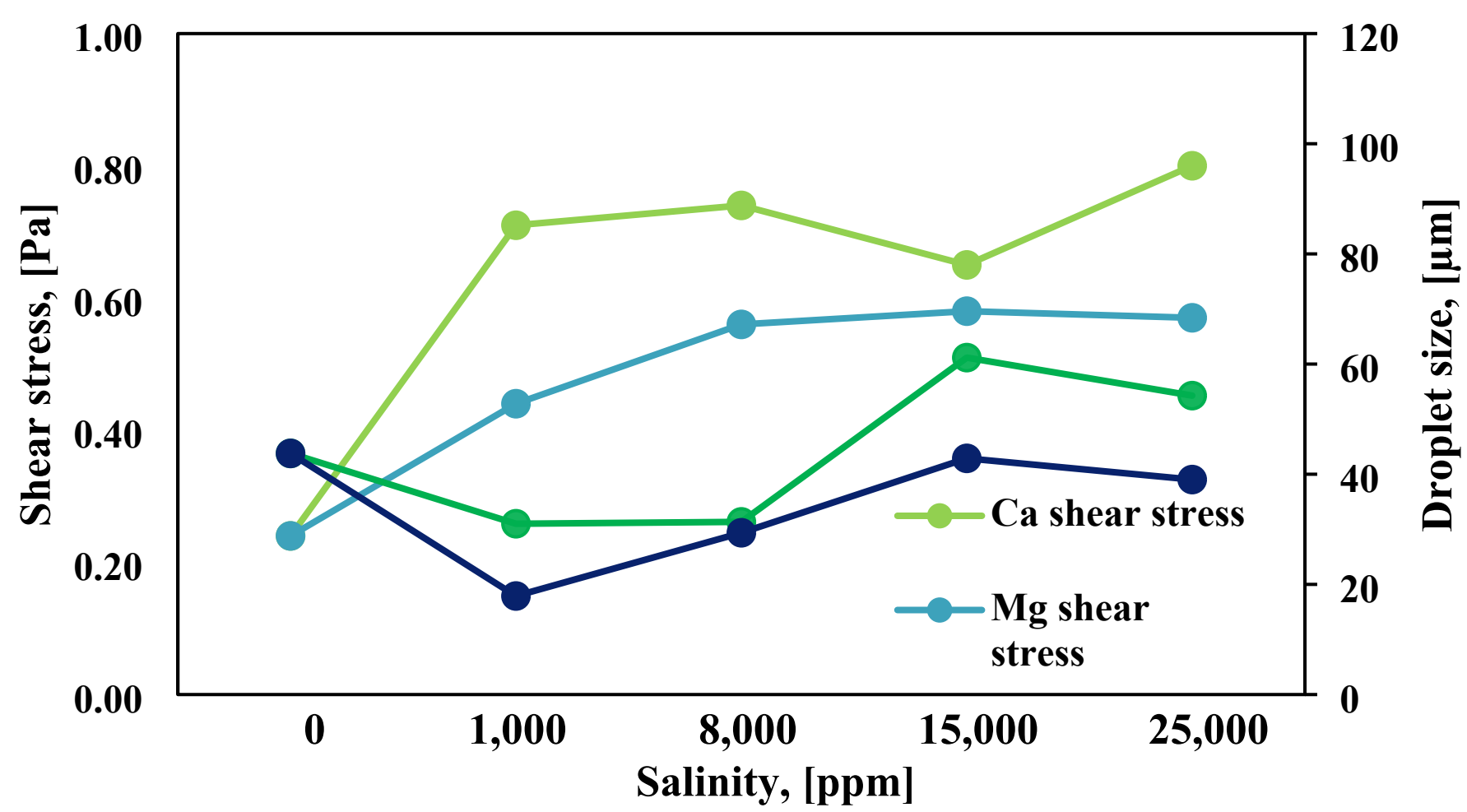

Figure S10. Shear stress and droplet size for emulsions prepared with $CaCl_2$ and $MgCl_2$ brines, surfactant (H) at concentrations of 5.0%v/v on day 7, with a shear rate of 4.17 s^{-1}.

ROJA program

Characterizing emulsions based on droplet size distribution presents significant challenges due to the morphological variability of the disperse phase. The statistical analysis of droplet size is essential for evaluating key physicochemical properties of emulsions, such as stability and viscosity. In this study, we analyzed 144 images (2592 × 1944 pixels), identifying a total of 20,842 droplets, with an average of 145 droplets per image. The droplet radii ranged from 3.71 to 4,984.85 μm, with a mean radius of 43.66 μm. From these measurements, comprehensive droplet size distributions were obtained to assess the dispersion characteristics of the system.

ROJA was developed in Python 3.7 using a combination of open-source libraries including OpenCV, NumPy, Matplotlib, Pandas, and scikit-image, among others. This tool enables automated identification and segmentation of droplets in microscopic images, allowing for statistical analysis of droplet size distributions, including radius, diameter, area, and count. Each image (2592 × 1944 pixels) was calibrated to a resolution of 2310 pixels per millimeter. ROJA outperforms standard tools such ImageJ by generating cleaner masks, reducing noise interference, and enabling high-throughput processing. The code is available in the supporting information.

CODE

```
%matplotlib widget
import cv2
import numpy as np
import matplotlib.pyplot as plt
import imageio.v3 as iio
import statistics as st
import pandas as pd
import math as mt
import ipympl
import skimage
import skimage.color
import skimage.transform
import skimage.util
import skimage.draw
import skimage.filters
import skimage.measure
import random
import glob


def Gotas(archivo, factor):

    # upload image
    #archivo = "30-70-1 25M2-75M1 dia 0.bmp"
    solution_image = iio.imread(uri=archivo)
    fig, ax = plt.subplots()
    plt.imshow(solution_image)

    gray_solution = skimage.color.rgb2gray(solution_image)

    blurred_image = skimage.filters.gaussian(gray_solution, sigma=1.0)

    pix_x = blurred_image.shape[0]
    pix_y = blurred_image.shape[1]
    pix_no = blurred_image.shape[0]*blurred_image.shape[1]
    pix_min = np.min(blurred_image)
    pix_max = np.max(blurred_image)
    pix_mean = np.mean(blurred_image)
    pix_median = np.median(blurred_image)
    pix_std = np.std(blurred_image)

    mask = blurred_image < np.mean(blurred_image) - (1/3)*np.std(blurred_image) #

    labeled_image, count = skimage.measure.label(mask, return_num=True)
```

```python
# compute object features and extract object areas
object_features = skimage.measure.regionprops(labeled_image)
object_areas = [objf["area"] for objf in object_features]
object_areas = np.zeros(len(object_areas)+1)
for i in range(labeled_image.shape[0]):
    for j in range(labeled_image.shape[1]):
        object_areas[labeled_image[i, j]] = object_areas[labeled_image[i, j]] + 1
aux = []
for i in range (len(object_areas)):
    if object_areas[i] > 14:
        aux.append(object_areas[i])
object_areas = np.array(aux)

min_area = int((3/5)*np.mean(object_areas[1:]))

aux = []
object_areas = [objf["area"] for objf in object_features]
object_areas = np.zeros(len(object_areas)+1)
for i in range(labeled_image.shape[0]):
    for j in range(labeled_image.shape[1]):
        object_areas[labeled_image[i, j]] = object_areas[labeled_image[i, j]] + 1
for i in range(len(object_areas)):
    if object_areas[i] < min_area:

        aux.append(i)

aux = np.array(aux)
for i in range(labeled_image.shape[0]):
    for j in range(labeled_image.shape[1]):
        if labeled_image[i, j] in aux:
            mask[i, j] = False

labeled_image, count = skimage.measure.label(mask, return_num=True)

# compute object features and extract object areas
object_features = skimage.measure.regionprops(labeled_image)
object_areas = [objf["area"] for objf in object_features]

object_areas = np.zeros(len(object_areas)+1)
for i in range(labeled_image.shape[0]):
    for j in range(labeled_image.shape[1]):
        object_areas[labeled_image[i, j]] = object_areas[labeled_image[i, j]] + 1

len1 = len(object_areas) - 1

mask = blurred_image > np.mean(blurred_image) - (1/3)*np.std(blurred_image) #

labeled_image, count = skimage.measure.label(mask, return_num=True)

# compute object features and extract object areas
object_features = skimage.measure.regionprops(labeled_image)
object_areas = [objf["area"] for objf in object_features]
object_areas = np.zeros(len(object_areas)+1)
for i in range(labeled_image.shape[0]):
    for j in range(labeled_image.shape[1]):
        object_areas[labeled_image[i, j]] = object_areas[labeled_image[i, j]] + 1
aux = []
for i in range (len(object_areas)):
    if object_areas[i] > 14:
        aux.append(object_areas[i])
object_areas = np.array(aux)
```

```
    min_area = int((3/5)*np.mean(object_areas[1:]))

    aux = []
    object_areas = [objf["area"] for objf in object_features]
    object_areas = np.zeros(len(object_areas)+1)
    for i in range(labeled_image.shape[0]):
        for j in range(labeled_image.shape[1]):
            object_areas[labeled_image[i, j]] = object_areas[labeled_image[i, j]] + 1
    for i in range(len(object_areas)):
        if object_areas[i] < min_area:

            aux.append(i)

    aux = np.array(aux)
    for i in range(labeled_image.shape[0]):
        for j in range(labeled_image.shape[1]):
            if labeled_image[i, j] in aux:
                mask[i, j] = False

    labeled_image, count = skimage.measure.label(mask, return_num=True)

    # compute object features and extract object areas
    object_features = skimage.measure.regionprops(labeled_image)
    object_areas = [objf["area"] for objf in object_features]

    object_areas = np.zeros(len(object_areas)+1)
    for i in range(labeled_image.shape[0]):
        for j in range(labeled_image.shape[1]):
            object_areas[labeled_image[i, j]] = object_areas[labeled_image[i, j]] + 1

    len2 = len(object_areas) - 1

    if len1 > len2:

        mask = blurred_image < np.mean(blurred_image) - (1/3)*np.std(blurred_image) #

        labeled_image, count = skimage.measure.label(mask, return_num=True)

        # compute object features and extract object areas
        object_features = skimage.measure.regionprops(labeled_image)
        object_areas = [objf["area"] for objf in object_features]
        object_areas = np.zeros(len(object_areas)+1)
        for i in range(labeled_image.shape[0]):
            for j in range(labeled_image.shape[1]):
                object_areas[labeled_image[i, j]] = object_areas[labeled_image[i, j]]
+ 1
        aux = []
        for i in range (len(object_areas)):
            if object_areas[i] > 14:
                aux.append(object_areas[i])
        object_areas = np.array(aux)

        min_area = int((3/5)*np.mean(object_areas[1:]))

        aux = []
        object_areas = [objf["area"] for objf in object_features]
        object_areas = np.zeros(len(object_areas)+1)
        for i in range(labeled_image.shape[0]):
            for j in range(labeled_image.shape[1]):
                object_areas[labeled_image[i, j]] = object_areas[labeled_image[i, j]]
+ 1
        for i in range(len(object_areas)):
            if object_areas[i] < min_area:
```

```
                aux.append(i)

        aux = np.array(aux)
        for i in range(labeled_image.shape[0]):
            for j in range(labeled_image.shape[1]):
                if labeled_image[i, j] in aux:
                    mask[i, j] = False

        labeled_image, count = skimage.measure.label(mask, return_num=True)

        # compute object features and extract object areas
        object_features = skimage.measure.regionprops(labeled_image)
        object_areas = [objf["area"] for objf in object_features]

        object_areas = np.zeros(len(object_areas)+1)
        for i in range(labeled_image.shape[0]):
            for j in range(labeled_image.shape[1]):
                object_areas[labeled_image[i, j]] = object_areas[labeled_image[i, j]]
+ 1

        got_min = np.min(object_areas[1:])
        got_max = np.max(object_areas[1:])
        got_mean = np.mean(object_areas[1:])
        got_median = np.median(object_areas[1:])
        got_mode = st.mode(object_areas[1:])
        got_std = np.std(object_areas[1:])
        r1 = mt.pow(got_min/mt.pi, 0.5)
        gota = object_areas[1:]
        for i in range(len(gota)):

            if gota[i] == got_min:

                gota[i] = r1/factor
            else:

                gota[i] = ((gota[i] - mt.pi*r1*r1)/(2*mt.pi*r1) + r1)/factor
        np.savetxt(archivo + '_gotas.csv', gota, delimiter=',')
        got_min = r1/factor
        got_max = ((got_max - mt.pi*r1*r1)/(2*mt.pi*r1) + r1)/factor
        got_mean = ((got_mean - mt.pi*r1*r1)/(2*mt.pi*r1) + r1)/factor
        got_median = ((got_median - mt.pi*r1*r1)/(2*mt.pi*r1) + r1)/factor
        got_mode = ((got_mode - mt.pi*r1*r1)/(2*mt.pi*r1) + r1)/factor

    else:
        mask = blurred_image > np.mean(blurred_image) - (1/3)*np.std(blurred_image) #

        labeled_image, count = skimage.measure.label(mask, return_num=True)

        # compute object features and extract object areas
        object_features = skimage.measure.regionprops(labeled_image)
        object_areas = [objf["area"] for objf in object_features]
        object_areas = np.zeros(len(object_areas)+1)
        for i in range(labeled_image.shape[0]):
            for j in range(labeled_image.shape[1]):
                object_areas[labeled_image[i, j]] = object_areas[labeled_image[i, j]]
+ 1
        aux = []
        for i in range (len(object_areas)):
            if object_areas[i] > 14:
                aux.append(object_areas[i])
        object_areas = np.array(aux)
```

```
        min_area = int((3/5)*np.mean(object_areas[1:]))

        aux = []
        object_areas = [objf["area"] for objf in object_features]
        object_areas = np.zeros(len(object_areas)+1)
        for i in range(labeled_image.shape[0]):
            for j in range(labeled_image.shape[1]):
                object_areas[labeled_image[i, j]] = object_areas[labeled_image[i, j]]
+ 1
        for i in range(len(object_areas)):
            if object_areas[i] < min_area:

                aux.append(i)

        aux = np.array(aux)
        for i in range(labeled_image.shape[0]):
            for j in range(labeled_image.shape[1]):
                if labeled_image[i, j] in aux:
                    mask[i, j] = False

        labeled_image, count = skimage.measure.label(mask, return_num=True)

        # compute object features and extract object areas
        object_features = skimage.measure.regionprops(labeled_image)
        object_areas = [objf["area"] for objf in object_features]

        object_areas = np.zeros(len(object_areas)+1)
        for i in range(labeled_image.shape[0]):
            for j in range(labeled_image.shape[1]):
                object_areas[labeled_image[i, j]] = object_areas[labeled_image[i, j]]
+ 1

        got_min = np.min(object_areas[1:])
        got_max = np.max(object_areas[1:])
        got_mean = np.mean(object_areas[1:])
        got_median = np.median(object_areas[1:])
        got_mode = st.mode(object_areas[1:])
        got_std = np.std(object_areas[1:])
        gota = np.power(object_areas[1:]/mt.pi, 0.5)/factor
        np.savetxt(archivo + '_gotas.csv', gota, delimiter=',')
        got_min = mt.pow(got_min/mt.pi, 0.5)/factor
        got_max = mt.pow(got_max/mt.pi, 0.5)/factor
        got_mean = mt.pow(got_mean/mt.pi, 0.5)/factor
        got_median = mt.pow(got_median/mt.pi, 0.5)/factor
        got_mode = mt.pow(got_mode/mt.pi, 0.5)/factor

    fig, ax = plt.subplots()
    plt.imshow(mask, cmap="gray")
    fig.savefig(archivo + '_mask.png')

    print("Gotas: ")
    print("No: ", len(object_areas) - 1)
    print("min: ", np.min(object_areas[1:]))
    print("max: ", np.max(object_areas[1:]))
    print("mean: ", np.mean(object_areas[1:]))
    print("median: ", np.median(object_areas[1:]))
    print("mode: ", st.mode(object_areas[1:]))
    print("std: ", np.std(object_areas[1:]))
    print(" ")
    got_no = len(object_areas) - 1

    # To remove small objects from the tagged image
    for object_id, objf in enumerate(object_features, start=1):
```

```
        if objf["area"] < min_area:

            labeled_image[labeled_image == objf["label"]] = 0

    object_areas = np.array([objf["area"] for objf in object_features])
    object_labels = np.array([objf["label"] for objf in object_features])
    small_objects = object_labels[object_areas < min_area]
    labeled_image[np.isin(labeled_image,small_objects)] = 0

    connectivity=2
    object_mask = skimage.morphology.remove_small_objects(mask,min_area)
    labeled_image, n = skimage.measure.label(object_mask, connectivity=connectivity,
return_num=True)

    colored_label_image = skimage.color.label2rgb(labeled_image, bg_label=0)
    fig, ax = plt.subplots()
    plt.imshow(colored_label_image)
    plt.axis("off");
    fig.savefig(archivo + '_colores.png')

    object_areas = np.array([objf["area"] for objf in
skimage.measure.regionprops(labeled_image)])
    object_areas = np.insert(0,1,object_areas)
    colored_area_image = object_areas[labeled_image]
    fig, ax = plt.subplots()
    im = plt.imshow(colored_area_image)
    cbar = fig.colorbar(im, ax=ax, shrink=0.85)
    cbar.ax.set_title("Area")
    plt.axis("off");
    fig.savefig(archivo + '_escala.png')

    return pix_x, pix_y, pix_no, pix_min, pix_max, pix_mean, pix_median, pix_std,
got_no, got_min, got_max, got_mean, got_median, got_mode, got_std

def Estadistica():

    caso = 0
    all_files = glob.glob("*_gotas.csv")
    for filename in all_files:

        if caso == 0:

            data = np.loadtxt(filename, delimiter=',')
            caso = 1
        else:
            aux = np.loadtxt(filename, delimiter=',')
            data = np.concatenate((data, aux))
    np.savetxt('GotasTotales.csv', data, delimiter=',')
    got_no = len(data)
    got_min = np.min(data)
    got_max = np.max(data)
    got_mean = np.mean(data)
    got_median = np.median(data)
    got_mode = st.mode(data)
    got_std = np.std(data)
    lista = []
    lista.append([got_no, got_min, got_max, got_mean, got_median, got_mode, got_std])
    lista = np.array(lista)
    lista_df = pd.DataFrame(lista, columns = ['got_no', 'got_min', 'got_max',
'got_mean', 'got_median', 'got_mode', 'got_std'])
    lista_df.to_csv('EstadisticaGotas.csv')

    return "listo"
```

```
# example of use
factor = 2310 # No. of pixels equivalent to 1 mm

lista = []
all_files = glob.glob("*.jpg")
for filename in all_files:

    pix_x, pix_y, pix_no, pix_min, pix_max, pix_mean, pix_median, pix_std, got_no,
got_min, got_max, got_mean, got_median, got_mode, got_std = Gotas(filename, factor)
    lista.append([filename, pix_x, pix_y, pix_no, pix_min, pix_max, pix_mean,
pix_median, pix_std, got_no, got_min, got_max, got_mean, got_median, got_mode,
got_std])

lista = np.array(lista)
lista_df = pd.DataFrame(lista, columns = ['nombre_imagen', 'pix_x', 'pix_y', 'pix_no',
'pix_min', 'pix_max', 'pix_mean', 'pix_median', 'pix_std', 'got_no', 'got_min',
'got_max', 'got_mean', 'got_median', 'got_mode', 'got_std'])

lista_df.to_csv('AnalisisCarpeta.csv')
```

Surfactant "H" Characterization

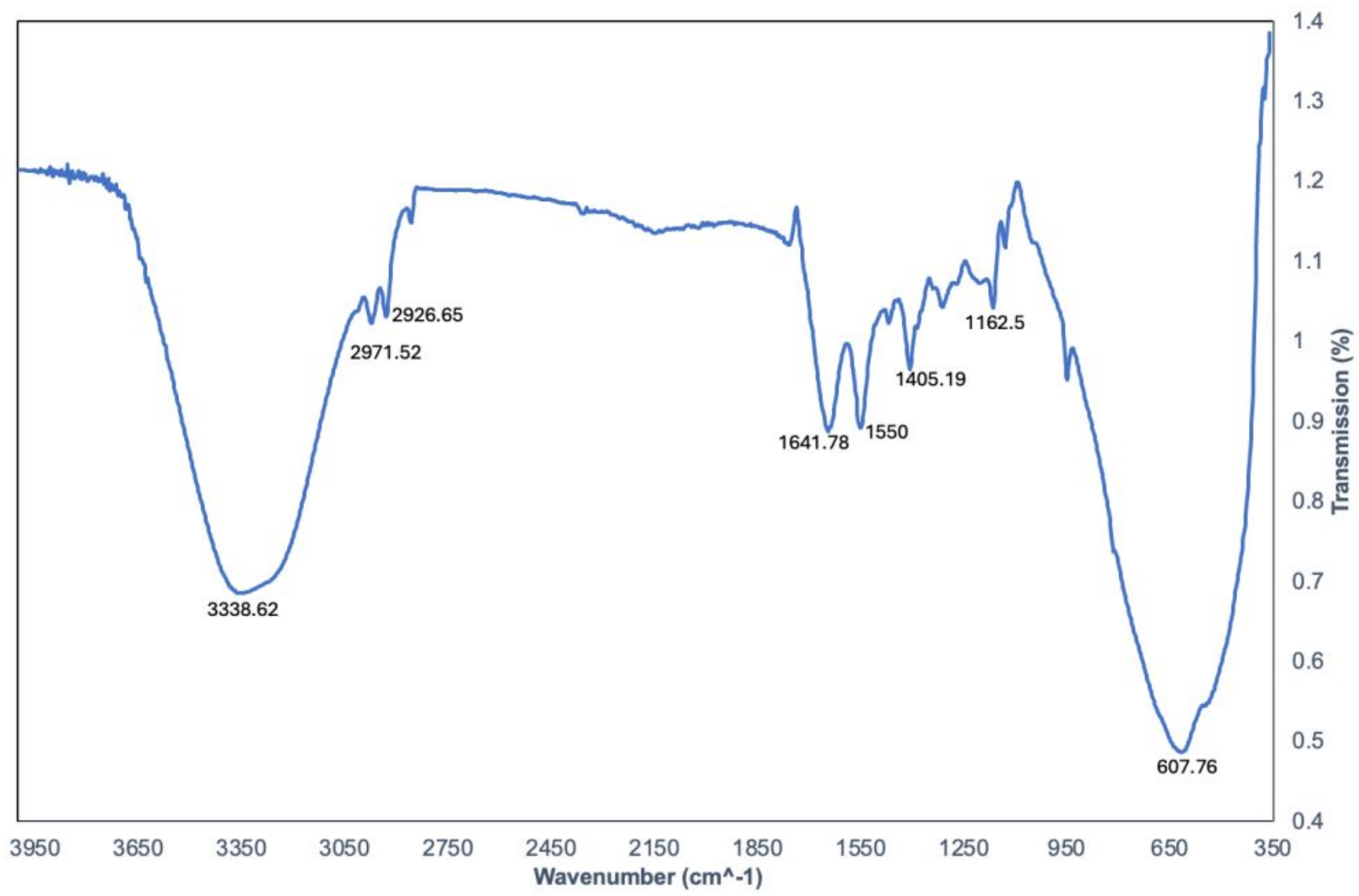

Figure S11. Anionic surfactant (H) Infrared Spectra

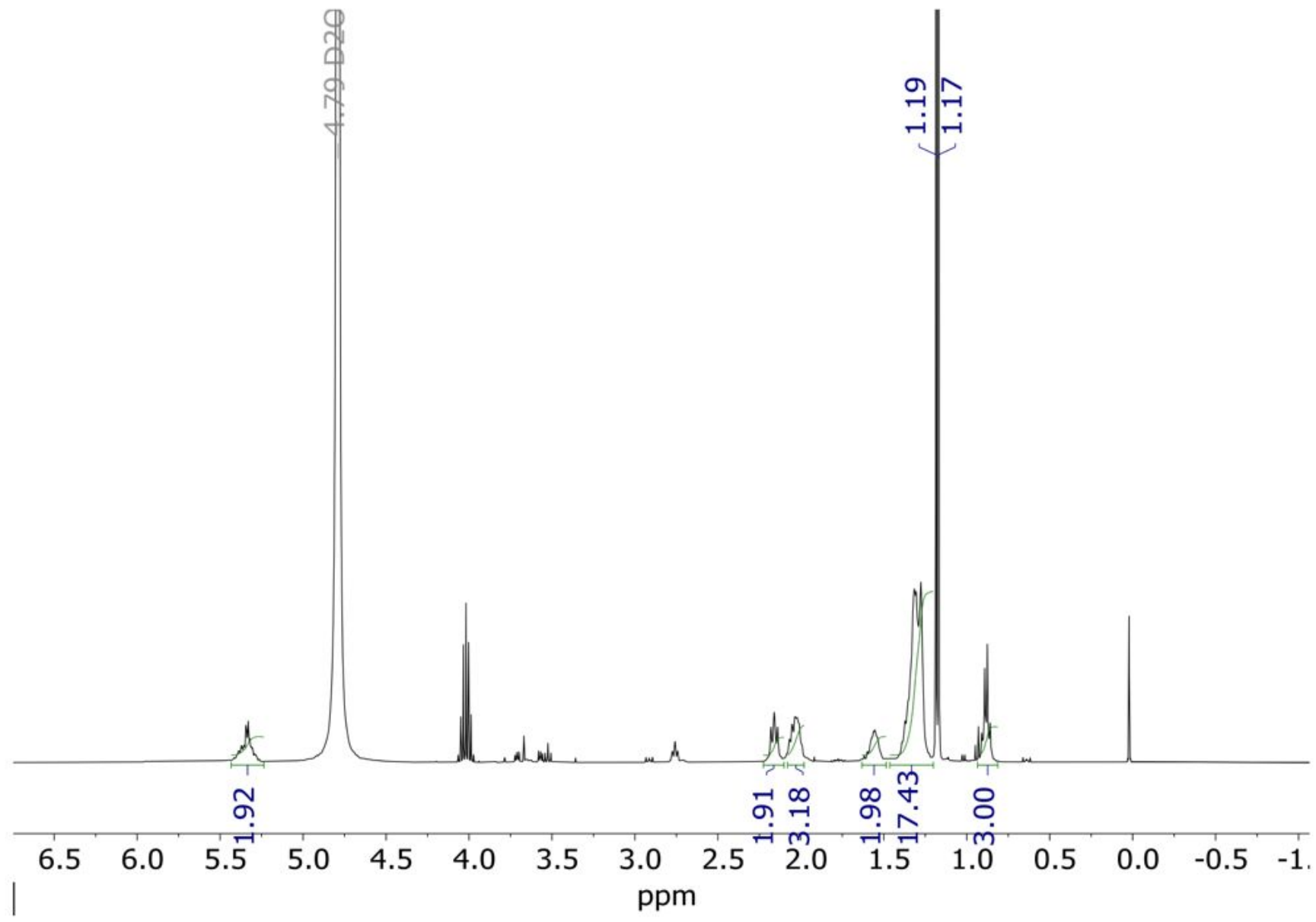

Figure S12. Anionic surfactant (H) ^{1}H NMR

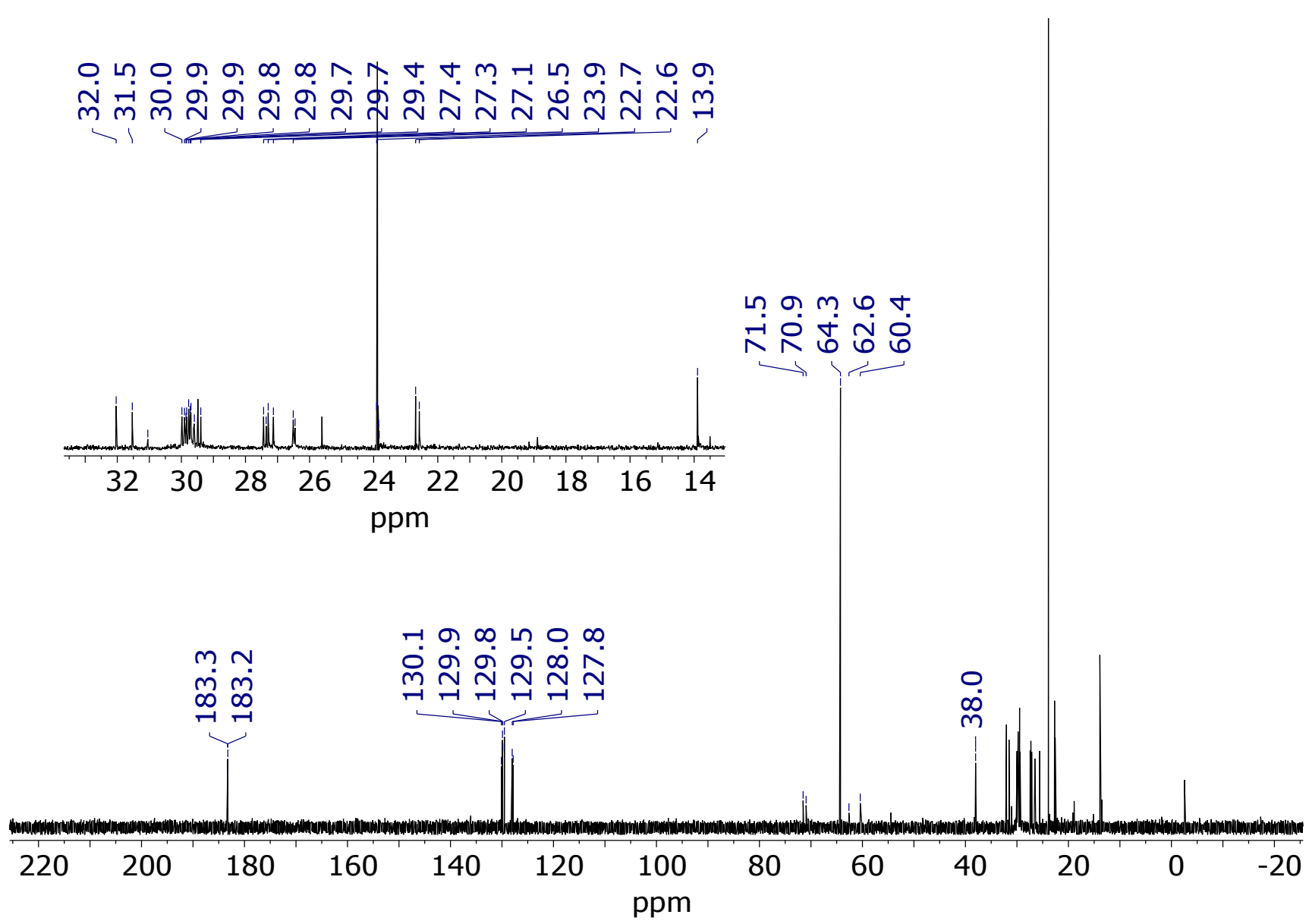

Figure S13. Anionic surfactant (H) ^{13}C NMR
